# Optimized NCFET-based approximate multiplier for energy-aware image applications

**DOI:** 10.1038/s41598-025-17805-5

**Published:** 2025-10-03

**Authors:** Mamidipaka B. R. Srinivas, Konguvel Elango

**Affiliations:** School of Electronics Engineering, Vellore Institute of Technology, Vellore, 632014 Tamil Nadu India

**Keywords:** Biomedical engineering, Electrical and electronic engineering

## Abstract

Applications for multimedia at high speeds has driven the need for error-resilient circuits that trade-off with accuracy while enhancing performance. However, the challenges posed by CMOS scaling are significant, and they have steered VLSI engineers towards exploring beyond-CMOS technology, Negative Capacitance FETs (NCFETs) with a sense of urgency. Enhancing the performance of multipliers is essential, as they are key components in various applications, including image processing and advanced learning algorithms. With the benefits of approximate computing for reducing the energy consumption of applications where exactness is not a requirement while simplifying the design of the circuits. The work here develops highly efficient approximate 4:2 compressors and multiplier circuits, representing core elements in approximate computing systems. This study introduces an innovative 8*8 approximate multiplier based on a 45nm Verilog-A NCFET technology model. It features a cutting-edge 4:2 compressor engineered for exceptional energy efficiency. By operating at a remarkably low voltage of 0.5V, the multiplier significantly reduces power consumption and energy usage compared to existing architectures. Our simulation results are compelling: the 4:2 compressor consumes just 0.122aJ of energy with NCFET technology, far outperforming alternatives like 31.67aJ, 10.49aJ, 172.75aJ in 45nm CMOS technology at 1V, which the proposed work outperforms them despite operating at half the voltage. Moreover, we have successfully realised an 8*8 approximate multiplier incorporating these compressors for image multiplication using MATLAB. The proposed design shows substantial improvements in PSNR and MSSIM and surpasses leading approximate accuracy and energy efficiency multipliers.

## Introduction

Over the past few years, the proliferation of microprocessors in battery-operated on-the-go-devices has prompted VLSI designers to pursue innovative new strategies to build more compatible and more power-efficient circuits. The ever-growing density and sophistication of nanoscale digital interconnects have resulted in to a significant increase in the power intensity and heat sinking of today’s VLSI chips. This increase in power intensity contributes to higher standby currents, deteriorating reliability and shorter IC life expectancy. In addition, energy consumption has emerged as a key challenge, especially for portable devices that rely on battery power^1^. Due to the mounting demand for processing data in real-time and the proliferation of data, there has been a marked acceleration in the development of high-performance computing systems^2^. Concurrently, advancements in semiconductor manufacturing and applications have precipitated rapid increases in the complexity, scale, and density of integrated circuits^3^. However, these advancements have concomitantly given rise to a substantial escalation in energy consumption, which has a deleterious effect on battery life and reliability^4^. Consequently, the reduction of energy usage has emerged as a paramount challenge in the realm of digital circuit design^5^. The factors influencing overall system power consumption include technology employed in manufacturing, the number of transistors, and scaled device technology^6^. Achieving a balance between these conflicting objectives, i.e., maximizing speed while minimizing energy consumption, remains a formidable challenge in the realm of VLSI design^3^.

Standard integrated circuits typically demand that accuracy be maintained throughout all operations. Hence, in many more lenient-error applications of multimedia, CNN^34^ exact arithmetic circuits gradually replaced approximations. Tolerance toward error forms part of various applications in general: multimedia generally comprises areas of image pattern recognition^33^, data mining processing and computer vision, areas heavily reliant upon human perception to execute, where quite often exactions toward stringent precision are forsaken. This simplifies hardware design, processes information more quickly, and uses significantly less energy. Stated differently, these domains have taken advantage of a compromise between accuracy and effectiveness. This approach is practical and resource-efficient because it works well for instances where even small computation errors are invisible to the user or only slightly impact functioning^7,8^.

Approximate computing offers a transformational technique to accelerate computations in error-tolerant applications while greatly boosting energy efficiency and simplifying circuit designs^9^. This design philosophy is highly adaptable, enabling its application across many levels of abstraction, including transistor-level optimisation, logic design, algorithm creation, architectural improvements, and software integration^10^. Accepting controlled error thresholds opens the door to creative solutions in systems with limited resources and performance.

Digital multipliers significantly affect system performance and are essential in applications like microprocessors, signal processing, encryption, and machine learning. They pose design issues due to their bulk and high power consumption, particularly on key lines^11^. A solution is provided by approximate computing, which lowers power usage while improving performance. Parallel designs provide speed at the expense of increased power consumption and possible hot spots, whereas sequential multipliers are slower but more power-efficient. For multiplier designs to be dependable and effective, power and speed must be optimized^12^. A typical multiplication process encompasses three distinct phases: 1. generating partial products through the use of AND gates, 2. reducing partial products, and 3. producing the final products via an adder structure^11^. Among these stages, the reduction of partial products stands out as the most crucial step with respect to energy consumption and spatial efficiency. To mitigate power consumption in the secondary stage, namely the partial product reduction stage, we adopted the notion of approximate computing with an exact compressor. This led to creating a 4:2 approximate compressor, which can be utilized in an 8*8 Dadda multiplier.

In this paper, we present a novel 4:2 compressor that utilizes only 10 transistors, significantly fewer than existing architectures. Our design employs NCFETs with a 45nm Verilog-A model in the Cadence Virtuoso tool, achieving a power consumption of 30.57nW and a delay of 4ps. This results in an energy efficiency of 0.122aJ. The 8*8 Dadda approximate multiplier, designed with this energy-efficient circuit, is applied in image processing applications. Evaluations using the PSNR and MSSIM indices for various images yielded values of 53.1dB and 0.9962, respectively, indicating that the designed 8*8 approximate multiplier achieves superior image quality.

## Related works

The paper discusses two 4:2 compressors tailored for multipliers, achieving a notable decrease in transistor count; design 2 employs 26 transistors, whereas the precise design uses 52. The approximate versions consume power at 217$\mu$W and 197$\mu$W, with energy efficiencies of 130 fJ and 112 fJ, respectively. The methodology involves comprehensive simulations assessing these compressors’ performance regarding power usage, delay, and transistor count, especially in the context of a Dadda multiplier. By leveraging computation imprecision, the designs attain significant metric reductions while keeping error rates within acceptable limits, as results indicate enhancements over precise designs across various performance measures. Nonetheless, this method has limitations, including an elevated error rate due to the simplified carry and sum outputs, potentially affecting final multiplication accuracy. For example, the first compressor exhibits a 37.5% error rate–lower than some existing solutions, yet this still creates hurdles for high-precision applications. Additionally, the balance between power savings and accuracy must be managed carefully, as these designs might not fit all application needs, especially those requiring stringent correctness. Design 1 has an error rate of 37.5% and showcases a PSNR exceeding 50 dB in image processing scenarios, reflecting a necessary trade-off between energy efficiency and accuracy^13^.

This paper outlines a methodology for designing and evaluating dual-quality 4:2 compressors for use in approximate Dadda multipliers. The focus is on creating a structure that enables toggling between approximate and exact operating modes, optimizing various performance hardware metrics . The design employs a blend of compressors, namely DQ4:$2C_{1}$ and DQ4:$2C_{4}$, to balance accuracy and design specifications. The effectiveness of these compressors is assessed through comparisons with existing approximate multipliers like U-ROBA, SSM8, and DRUM6, revealing considerable improvements in performance metrics. Nonetheless, limitations arise due to the necessary balancing precision with processing speed, as the approximate mode may yield a higher error rate than the exact mode, potentially affecting applications that demand high precision. The design’s complexity may also pose challenges in practical implementations, especially concerning integration and power management^12^.

This paper discusses a methodology for designing approximate compressors, particularly 4:2 and 5:2 types, aimed at boosting the performance of Dadda multipliers in multiplication tasks. The research introduces three innovative 4:2 and two 5:2 approximate compressors, which are incorporated into the development of approximate 8*8 Dadda multipliers. These designs aim to decrease the number of partial product stages, enhancing power efficiency and minimizing delay for energy-efficient VLSI architectures. Synthesis results show that these new designs significantly outperform existing ones regarding power, delay, and accuracy. However, challenges include a fundamental balancing precision and power efficiency, as approximate computing can lower accuracy for some applications. Furthermore, these designs might not be suitable for all kinds of multipliers, which could restrict their effectiveness in particular situations^14^. The proposed methodology focuses on creating innovative approximate compressors that adopt a distinctive method to reduce error probability and average error, while ensuring the output weight matches that of the input. This contrasts with conventional compressors that have carry outputs with double the weight. These compressors are incorporated into approximate multipliers to enhance electrical performance, facilitating a trade-off between precision and power consumption. The implementation involves designing various binary multipliers (8 * 8, 12 * 12, 16 * 16, and 20 * 20) using these compressors at different stages of partial product matrix (PPM) reduction. Furthermore, utilizing approximate compressors may introduce greater complexity in circuit design and pose challenges in maintaining the desired balance between performance and accuracy^15^.

The proposed methodology centres on creating an approximate multiplier using a modified 4:2 compressor and an error recovery module. This design seeks to improve multiplication operations in digital signal processors by minimizing hardware space, power use, and delay compared to conventional exact multipliers. Notably, the approximate multiplier only approximates the multiplication process, ensuring that all other operations remain precise, thus striking a balance between performance and accuracy. Results show that this design achieves a reduction of 23.2% to 24.4% in hardware area, 22.4% to 24.5% in power consumption, and a delay that is 11.2% to 17.0% shorter than that of traditional exact multipliers, along with an 11.7% enhancement in the mean error distance (MED) of multiplication outcomes compared to earlier designs. Nonetheless, the method has limitations, including a possible reduction in the accuracy of the multiplication results due to the nature of approximation, which may not be appropriate for all high-precision applications. Furthermore, while the error recovery module can address some inaccuracies, it could also lead to added design complexity and overhead^16^.

This work focuses on designing and analysing an approximate 4:2 compressor, which is integrated into an enhanced Dadda Multiplier architecture to improve efficiency and lower error rates. The approach involves leveraging the approximate compressor to decrease the output bit count, thereby minimizing input bits for later compressors in the multiplier framework. Such a strategy facilitates parallel computation, which is advantageous for multi-bit tasks, leading to notable enhancements in power consumption (reduced by 39%), processing speed (49% faster), and area (21% smaller) in comparison to precise designs. The accuracy of the proposed systems is assessed using error metrics, indicating an error rate of merely 12.5%, significantly lower than other current approximate compressors. Nevertheless, there are drawbacks, including the risk of diminished accuracy when substituting exact blocks with approximate ones in the most significant bit (MSB) columns, which could greatly affect output quality. Furthermore, the balance between power savings and accuracy requires careful consideration, as the design’s success may differ based on the specific application and the level of approximation employed^7^.

The proposed article focuses on designing an approximate multiplier that utilises current mode technology, specifically using 4:2 and 5:2 compressors made with carbon nanotube field effect transistors. (CNTFETs). The process encompasses three key steps: first, transforming input currents into multi-level voltages; second, establishing a voltage level detector; and third, producing output currents based on a set threshold voltage. This method seeks to decrease power consumption, delay, and the number of hardware units as opposed to present architectures. Simulation results reveal that the power delay product (PDP) for the 4:2 and 5:2 compressors is 0.0097 and 0.0131 fJ, respectively, indicating substantial gains in efficiency. Furthermore, the design of an 8 $\times$ 8 multiplier using these compressors shows improved performance regarding PSNR and MSSIM in image processing applications^17^.

This paper presents an approximate multiplier design that improves energy efficiency while maintaining acceptable accuracy for error-tolerant applications, such as artificial neural networks (ANNs) and multimedia processing using 7nm Tri-gate FinFET. The design features a partial product reduction unit combining exact and approximate 4:2 compressors and an error compensation module (ECM) to address inaccuracies. The architecture optimizes hardware overhead, delay, and power consumption, balancing performance and efficiency. The results show that the proposed multipliers achieve lower power-delay products (PDP) while retaining high accuracy in error-resilient applications^18^. NCFETs are much more power-efficient than CMOS^32^, CNTFET^17^, FinFET^18^, and SJT/MJT technologies. NCFETs utilize a ferroelectric material in the gate stack to create a negative capacitance effect and provide lower voltage and power consumption. This reduces dynamic and static power dissipation to a great extent, making NCFETs very energy-efficient. Although CMOS transistors are constrained by the subthreshold swing of 60 mV/decade, and CNTFETs, FinFET, and SJT/MJT technologies are constrained by fabrication and scalability, NCFETs can have subthreshold swings below this, allowing additional voltage scaling without impacting performance. This ease of integration with normal CMOS processes and synergy of low power consumption and high performance make NCFETs an appealing transistor technology for energy-efficient computing.

The^39^ presents two approximate 4:2 compressors designed using 45nm CMOS technology, the proposed work adopts a transistor level approach using NCFETs, resulting in several tangible benefits. The proposed design achieves a delay of 10ps, consumes only 0.269$\mu$W of power, and yields a PDP of 2.69aJ, utilizing just 10 transistors across 2 gate count. In contrast, the most efficient design in the^39^ reports 38ps delay, 0.295$\mu$W power, and a PDP of 11.21aJ, with a complexity of 54 transistors and 10 logic gates. These differences translate into approximately 3.8$\times$ lower delay, 4$\times$ improvement in PDP, and a 5$\times$ reduction in hardware in our design. Furthermore, our circuit maintains accuracy, achieving an MRED of $3.04 \times 10^{-2}$, comparable to their best result of $3.2 \times 10^{-2}$. A key distinction of our implementation is its ability to function reliably at 0.5V, where performance further improves to 4ps delay, 30nW power, and 0.12aJ PDP, taking advantage of the steep subthreshold behavior of NCFETs something not explored in^39^. These differences in energy efficiency, hardware cost, and low-voltage operation clearly underscore the uniqueness and practical value of the proposed method. The^40^ employs a FinFET-based implementation using 7nm technology Fin height and gate length, which are set to 18nm and 11nm, respectively, for both p-type and n-type transistors. While^40^ work presents useful architectural insights, a direct comparison with our design is not entirely appropriate due to the fundamental differences in transistor technology and fabrication node. Theproposed work is based on planar NCFET devices modeled at the 45nm node, simulated at the transistor level in Cadence Virtuoso using a Verilog-A model. As the performance metrics in FinFETs–particularly delay, leakage, and energy efficiency–are inherently influenced by their 3D geometry and sub-20nm scaling, juxtaposing them with NCFET results at 45nm without proper normalization may lead to biased conclusions

The remaining part of the paper focuses on introducing NCFET and its characteristics, highlighting its metrits in “Background of NCFET”. Section “Compressors” delves into compressors, including existing designs and the proposed 4:2 compressor. Section “Proposed 8*8 approximate multiplier” discusses the implementation of the proposed 4:2 compressor in an 8*8 Dadda approximate multiplier. Section “Accuracy analysis of the proposed design” covers the accuracy metrics in image processing applications. Finally, “Conclusion” discusses future directions and concludes the paper.

## Background of NCFET

In the last couple of decades, the continuous miniaturization of complementary metal-oxide-semiconductor (CMOS) technology has significantly transformed the field of information processing. This trend, famously captured in Moore’s Law by Gordon Moore, estimated that the transistor density in ICs would double every two years^19^. Unfortunately, scaling down the supply voltage proportionally was constrained by the fundamental limitations of device operating principles, creating a bottleneck for developing ultra-low-power electronic devices. Lowering the supply voltage is a highly effective method for addressing this issue, primarily because the power factor depends quadratically on the supply voltage. However, in the case of conventional MOSFETs, aggressive scaling of the supply voltage ($V_{DD}$) encounters fundamental constraints, notably referred to as the ’Boltzmann Tyranny,’ which limits reductions to around 60 mV per decade^20^. To overcome this limitation in conventional transistors, a range of devices has been developed called negative capacitance field-effect transistors (NCFET)^21^ have emerged as a promising solution to surpass the 60mV/decade subthreshold swing limitation by leveraging the voltage amplification effect provided by an embedded ferroelectric layer. NCFETs highly suitable for energy-efficient digital designs, including the approximate arithmetic architectures presented in this study. The device modify electron transport to surpass the minimum threshold of 2.3KT/q (where k is the Boltzmann constant, T is temperature, and q is charge), ensuring that the body factor ($V_{s}$) remains less than 1 as shown in Eq. (1).

**Figure 1:**
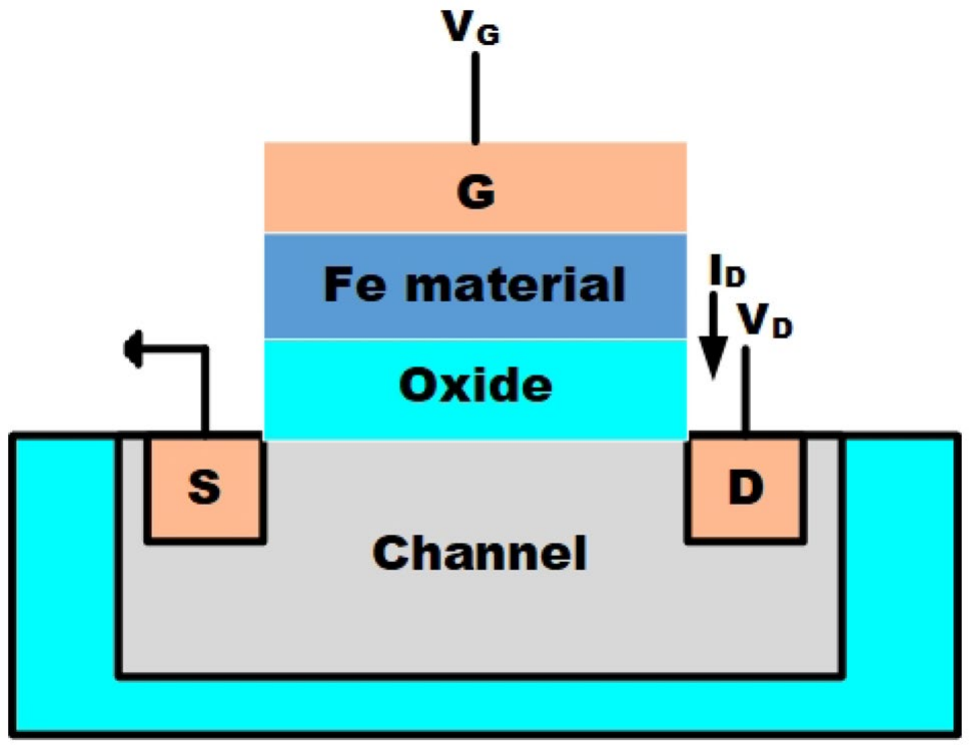
Device internal architecture.

**Figure 2:**
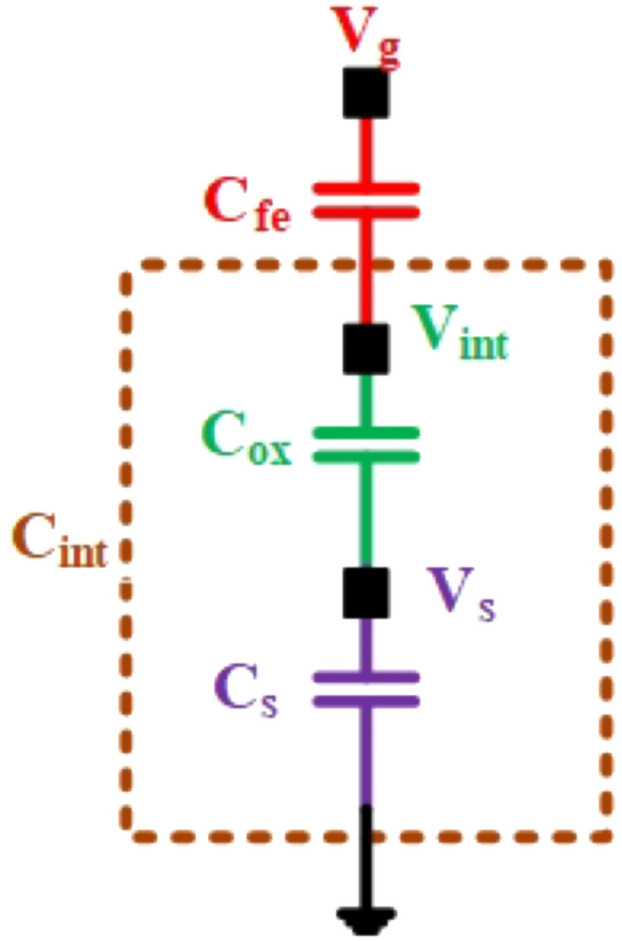
Equivalent capacitor model.

**Figure 3:**
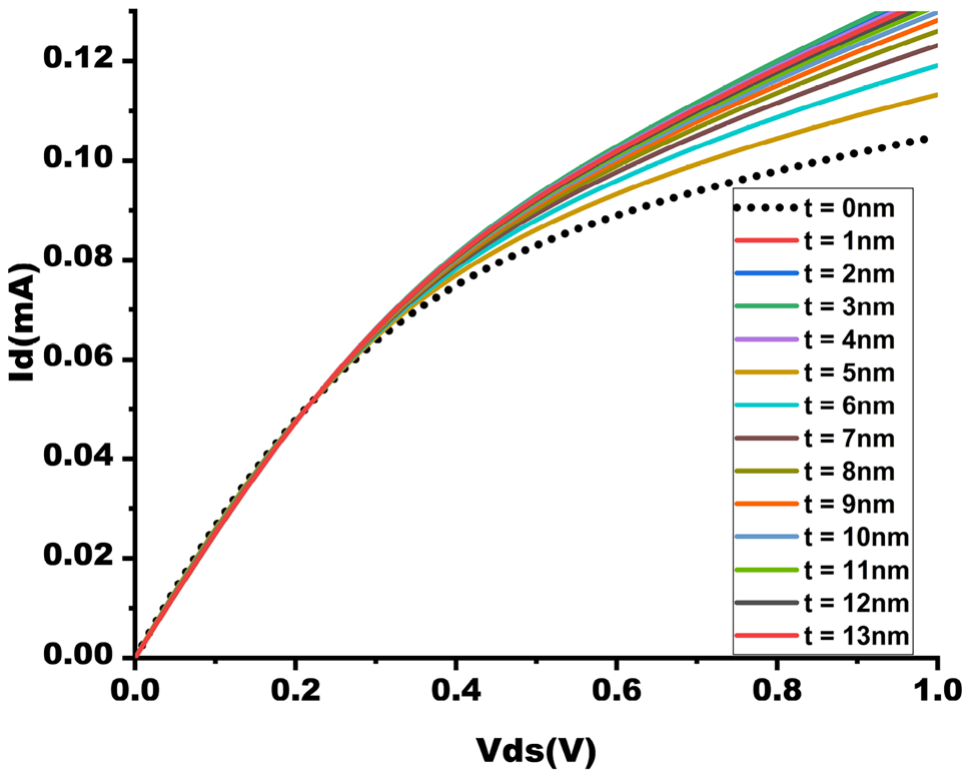
$I_{D}$-$V_{ds}$ showcasing the enhancement of ON current by varying $t_{fe}$ 0nm - 13nm.

The NCFET model employes in this study is using the Intel 40 nm p-type/n-type bulk FET as a reference Si-MOSFET^22^. This streamlined model, known as the MIT Virtual Source Ferroelectric (MVSNC) model, captures various distinct attributes of NCFETs^23^. The NCFET-based device integrates a ferroelectric (FE) material within the gate stack of a standard FET, as illustrated in Fig. 1. This incorporation leads to the amplification of the internal voltage of the benchmark transistor by the FE layer, as depicted in the analogous internal circuit representation in Fig. 2. and it is represented in Eq. (2). This significantly improves the $I_{on}$/$I_{off}$ ratio and an enhancement of the sub-threshold slope (SS), as depicted in Fig. 3 which preserving from switching speed.

1 $$\begin{aligned} subthreshold slope = \frac{\partial V_{g}}{\partial log_{10}I_{d}} = \frac{\partial V_{g}}{\partial V_{int}} \frac{\partial V_{int}}{\partial V_{s}} \frac{\partial V_{s}}{\partial log_{10}I_{d}} \end{aligned}$$

2 $$\begin{aligned} A_{v}= & \frac{\left| C_{fe} \right| }{\left| C_{fe} \right| -C_{int}} \end{aligned}$$

3 $$\begin{aligned} C_{g}= & \frac{\left| C_{fe} \right| C_{int}}{\left| C_{fe} \right| - C_{int}} \end{aligned}$$

**Figure 4:**
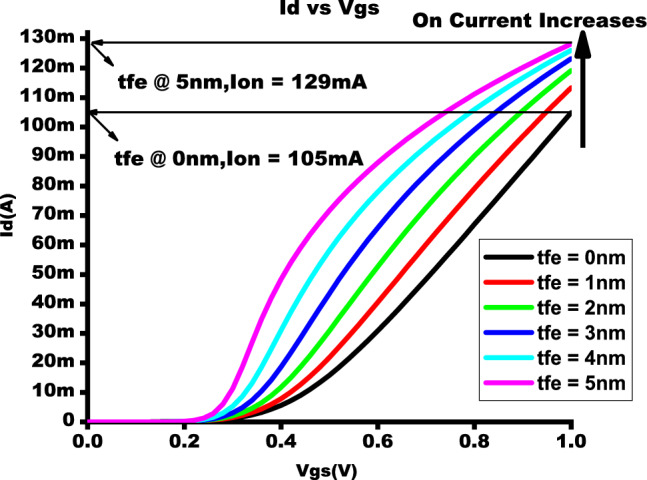
$I_{D}$-$V_{gs}$ showcasing the enhancement of ON current by varying $t_{fe}$ 0nm - 5nm.

**Figure 5:**
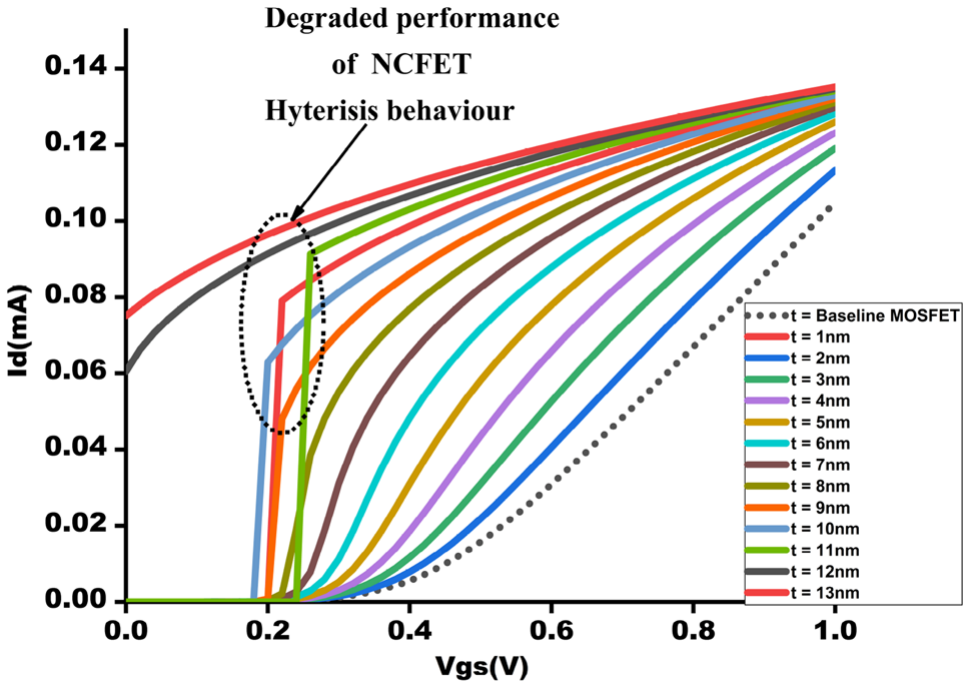
$I_{D}-V_{gs}$ showcasing the degraded behaviour by varying $t_{fe}$ 0–13 nm.

**Figure 6:**
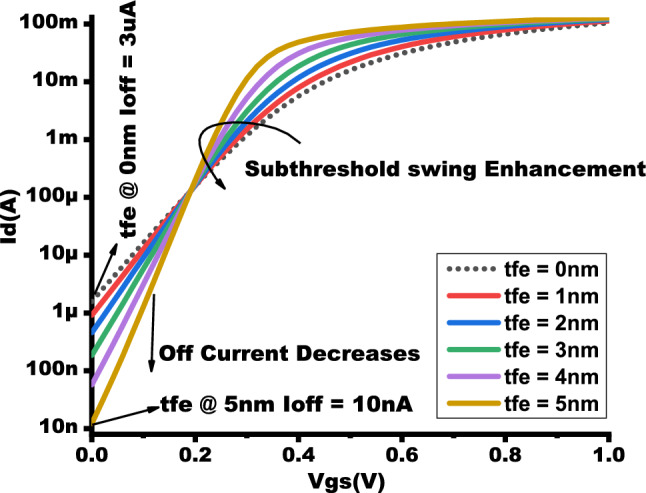
OFF current by varying $t_{fe}$ 0–5 nm.

**Figure 7:**
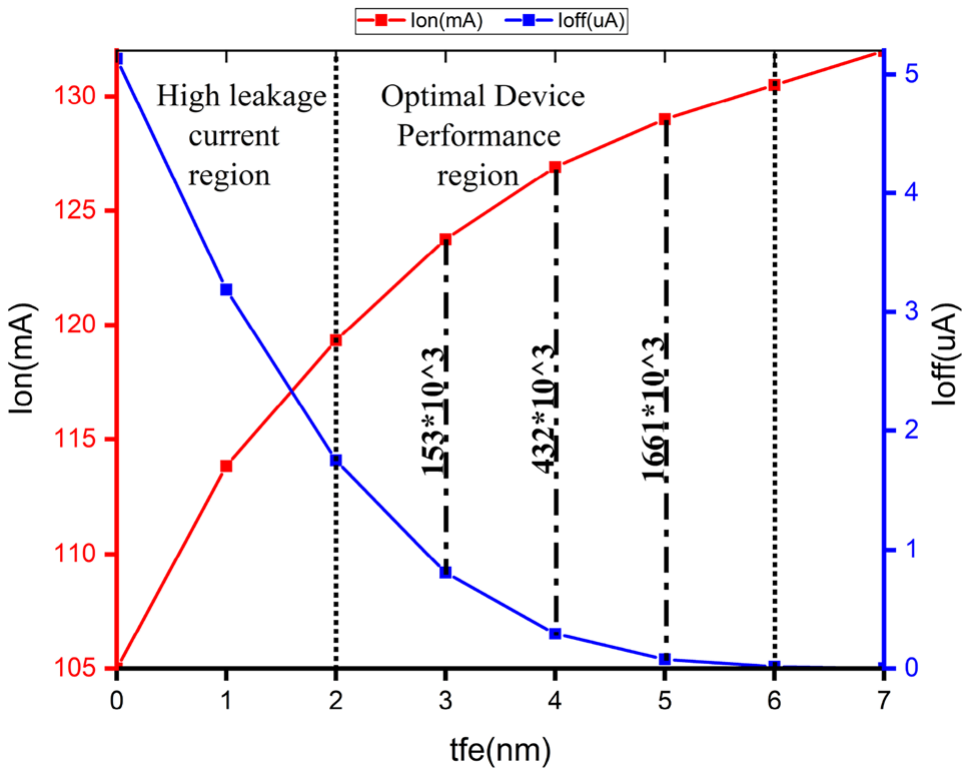
ON and OFF current attributes.

**Figure 8:**
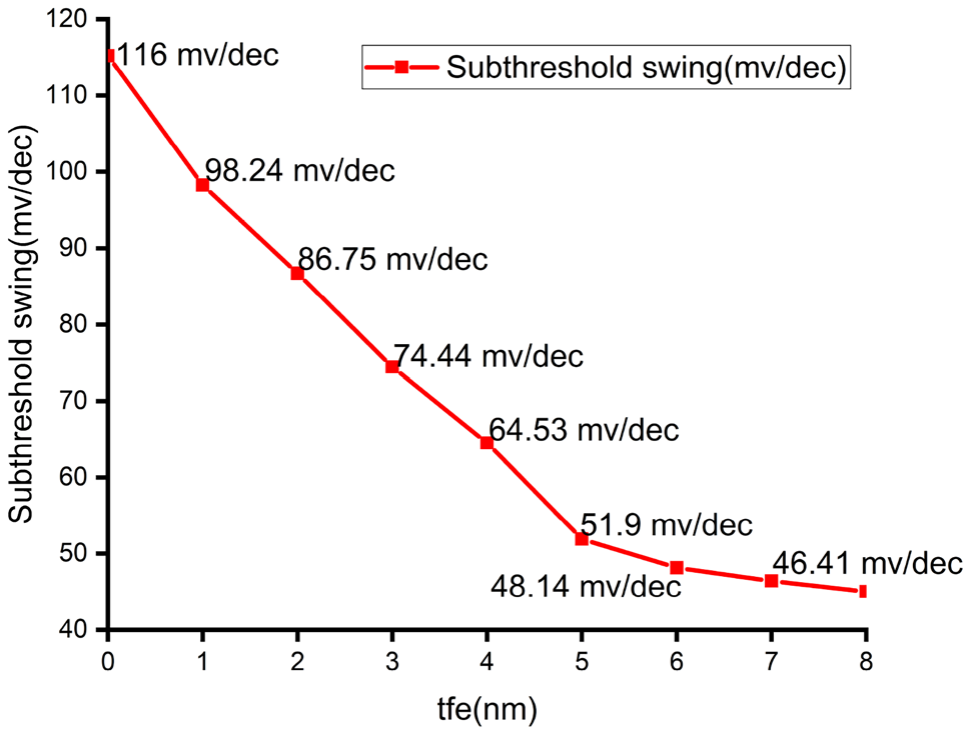
Subthreshold swing of NCFET varying $t_{fe}$ 0nm - 7nm.

**Figure 9:**
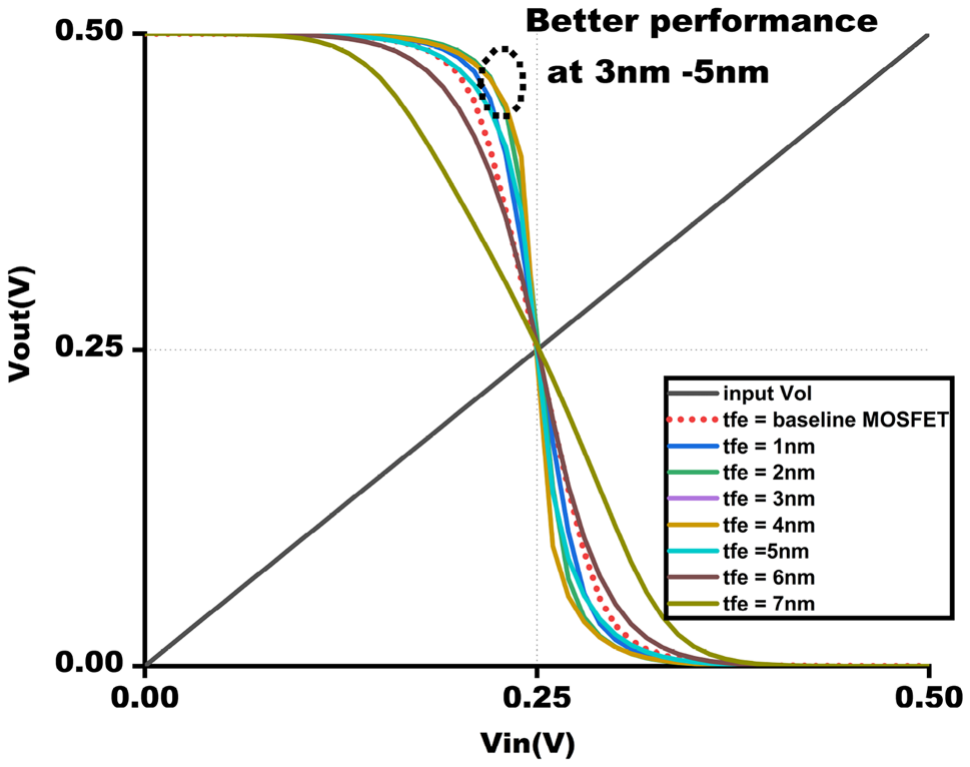
VTC @ $V_{gs}$ = 0.5 V varying $t_{fe}$ from 0 - 5nm.

**Figure 10:**
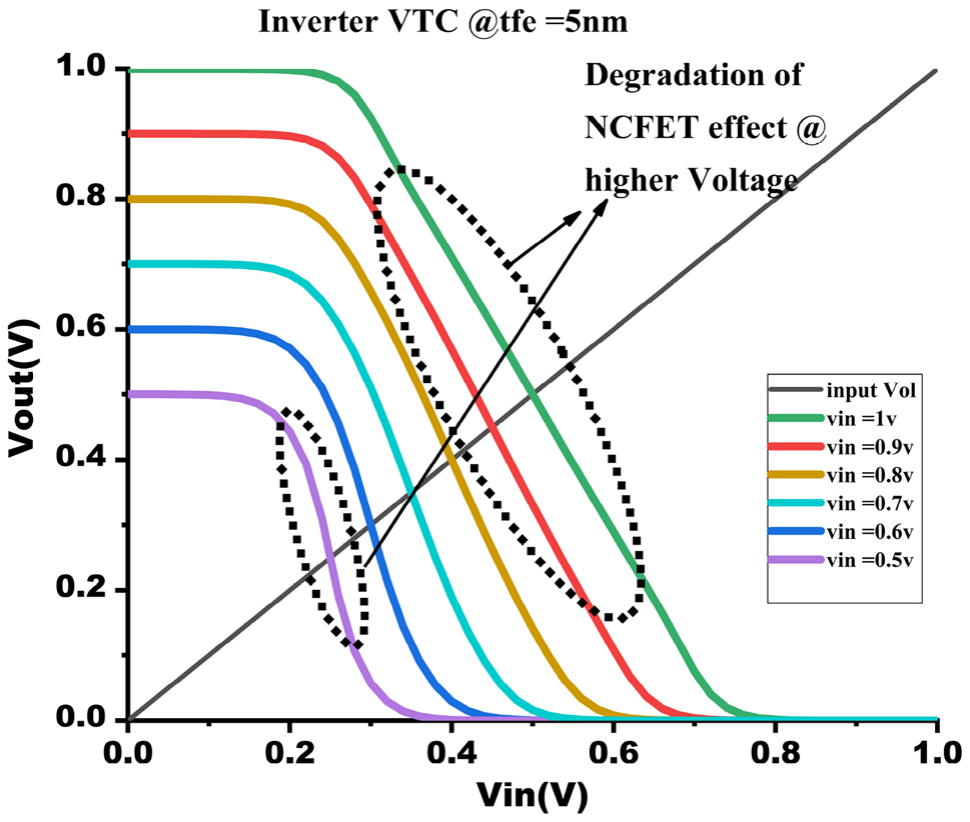
VTC @ $t_{fe}$ = 5 nm varying $V_{gs}$ from 0.5V - 1V.

To ensure from hysteresis-free operation, $\big |C_{fe} \big |$ must be greater than $C_{int}$, as derived from Eq. (3).To prevent this, the thickness of the ferroelectric layer ($t_{fe}$) should be adjusted.Therefore,it is crucial for achieving higher gain and hysteresis-free operation, essential for energy-efficient NCFET logic and operation. In overall NCFETs are steadily becoming the preferred choice over FinFET, CNTFET, and TFET technologies. NCFETs can reduce power consumption by up to 34$\%$ compared to FinFETs, all while maintaining their performance levels^35^. In high-density computing setups such as Google’s TPU, NCFETs have shown to be 2.8 times more efficient than traditional FinFETs by significantly cutting down cooling expenses, a critical factor in such environments^36^. The adaptable design of NCFETs enhances their ON/OFF current ratios and sub-threshold swing, even at reduced supply voltages, leading to significant improvements in overall device performance^37^. NCFETs excel in low-power applications, such as microwave wireless power transmission, where they demonstrate far superior rectification efficiency compared to traditional MOS devices^38^. This allows low power high performance logic blocks such as approximate compressors,multipliers as shown in this work.

### NCFET I–V characteristics

The I-V attributes of NCFETs are greatly influenced by the thickness of the feature $t_{fe}$ as shown in below figures. In Fig. 3 represents the graph of impact of Drain current $I_{D}$ when the input voltage is varying, i.e $v_{ds}$ from 0-1V. For $t_{fe}$ values from 3nm to 5nm, a balanced trade-off is observed between power efficiency and performance.

Figure 4 shows that NCFET exhibits improved ON current with Ion of 105mA at baseline MOSFET and 130mA at $t_{fe}$ 5nm, which has a 19.23 % augmentation of ON current for NCFET device and less subthreshold swing (SS) due to the negative capacitance effect, as shown in Fig. 6 with an OFF current of 10nA at $t_{fe}$ 5nm which performs with very low OFF current when compared to $t_{fe}$ 0nm of 3uA .As $t_{fe}$ increases to 13nm, NCFET displays hysteresis attribute, as seen in Fig. 5. This hysteresis occurs when the $C_{fe}$ becomes less than the internal baseline FET capacitance ($C_{int}$) leading to destabilizing of device. Figure 7 indicates that as $t_{fe}$ increases from 0nm to 7nm, NCFET ON current rises and leakage current decreases. For $t_{fe}$ values between 3nm and 5nm, NCFET shows a remararkable switching current ratio. However, beyond 5nm, the device displays hysteresis and degraded performance. Additionally, NCFET with 5nm $t_{fe}$ exhibits a significantly better subthreshold swing (51.9 mV/dec) than the baseline MOSFET (116 mV/dec), which shows a 55.25% reduction in the subthreshold swing when compared to baseline MOSFET as shown in Fig. 8. The analysis concludes justifies our design choices and ot opearting conditions in subsequent circuit level simulations.

### NCFET-based inverter VTC characteristics and transient analysis

**Figure 11:**
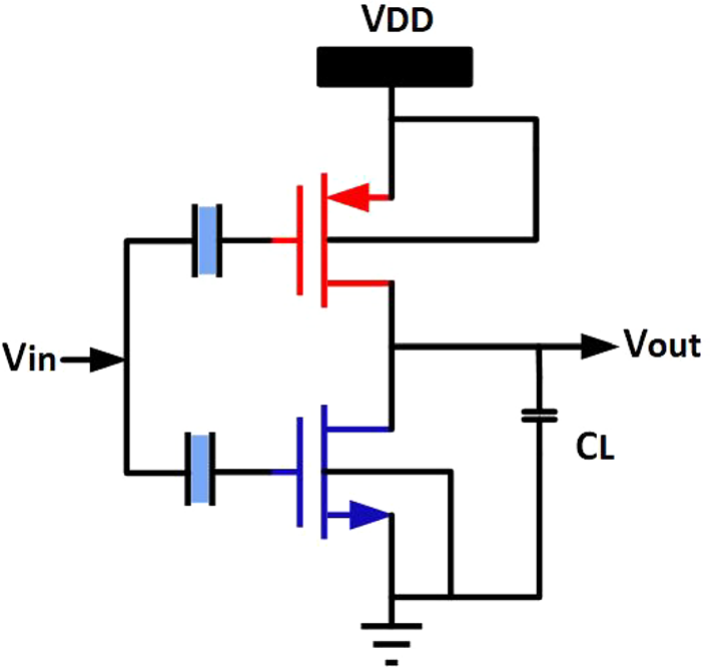
NCFET inverter.

The Digital Inverter is implemented using the NCFET with a technology node of 45nm as shown in Fig. 11, operated by varying voltages from 0.5V to 1V. The VTC are observed by connecting load capacitance to the inverter. Figure 10 shows the DC transfer attributes of the inverter by maintaining $t_{fe}$ at 5nm under varying varying voltage, which tells us that at high voltages, it losses an NCFET behaviour w.r.to $t_{fe}$ it is because of the ferroelectric layer, usually $HfZro_{x}$, poses challenges in NCFETs. These leads to depolarization, breakdown, and loss of negative capacitance,threshold shifts, and increased leakage current, undermining energy efficiency. Additionally, excessive drain-induced barrier lowering (DIBL) worsens subthreshold leakage, further lowering efficiency.So, it is preferred for the best to use NCFET for low-power applications, usually at 0.5V, which enables researchers to use applications like 8*8 approximate multipliers and beyond. Figure 9 shows the VTC attributes of the NCFET inverter operated at 0.5V by gradient thickness of ferroelectric oxide, i.e. $t_{fe}$ @ 0nm to 7nm, shows the improved performance compared to $t_{fe}$ = 0nm, which is normal conventional MOSFET. Moreover, increasing the thickness to up to 5nm improves the VTC of NCFETs. However, when the thickness exceeds 5nm, the VTC of the NCFET inverter deviates from the estimated performance because of a decline in the steep switching characteristics of NCFETs.

The evaluation of the performance metrics of intricate designs is shown in Table 1.

**Table 1:** NCFET-based combinational circuits operated at 0.5V.

S. no.	Design	tpdr (ns)	tpdf (ns)	Avg delay (ns)	Power consumption (nW)	PDP (aJ)	EDP (aJ ns)
1	Inverter	0.005	0.005	0.005	8.24	0.0412	0.0002
2	AND$_{2\text {input}}$	0.016	0.02	0.018	27.72	0.498	13.9
3	OR$_{2\text {input}}$	0.014	0.012	0.013	18	0.234	4.212
4	NOR$_{2\text {input}}$	0.005	0.004	0.0045	10.58	0.047	0.497
5	OR$_{3\text {input}}$	0.001	0.018	0.0095	18.32	0.174	3.18
6	NOR$_{3\text {input}}$	0.007	0.006	0.0065	10.97	0.0713	0.782
7	XOR$_{2\text {input}}$	0.017	0.017	0.017	45.99	0.781	35.918
8	Half adder$_{1\text {bit}}$	0.011	0.01	0.011	61.16	0.642	39.26
9	Full adder$_{1\text {bit}}$	0.01	0.01	0.01	220.2	2.2	484.44

The inverters have an impressively low average delay of just 0.005ns and consume only 8.24nW of power. Consequently, they achieve an exceptionally low Power Delay Product (PDP) of 0.0412 aJ and Energy Delay Product (EDP) of 0.0002 aJ $\cdot$ ns. Despite higher power consumption, components such as the XOR$\_$2input and Full Adder$\_$1bit maintain efficient PDP and EDP values. This highlights the potential of NCFETs for high-performance, energy-efficient applications with a thickness of 5nm; NCFETs demonstrate superior performance metrics, making them a compelling choice for low-power, high-speed electronic devices.

## Compressors

**Figure 12:**
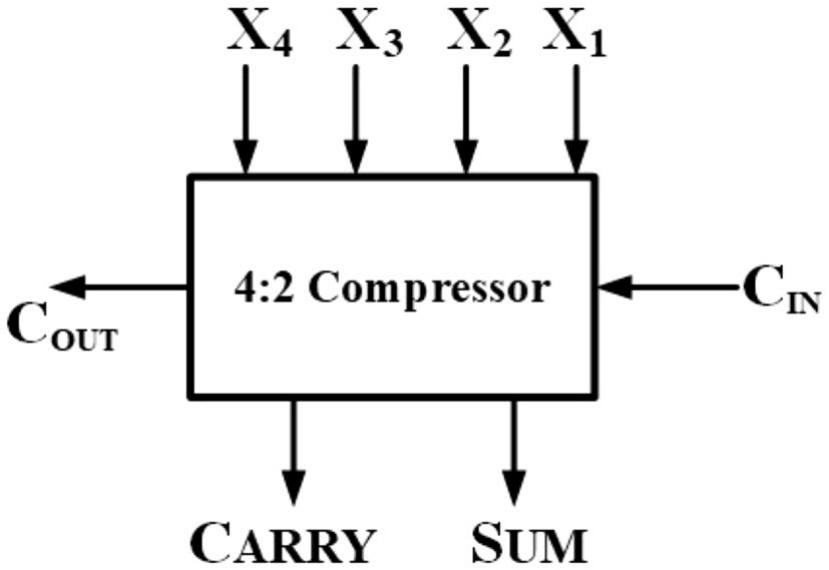
Block diagram of exact compressor.

**Figure 13:**
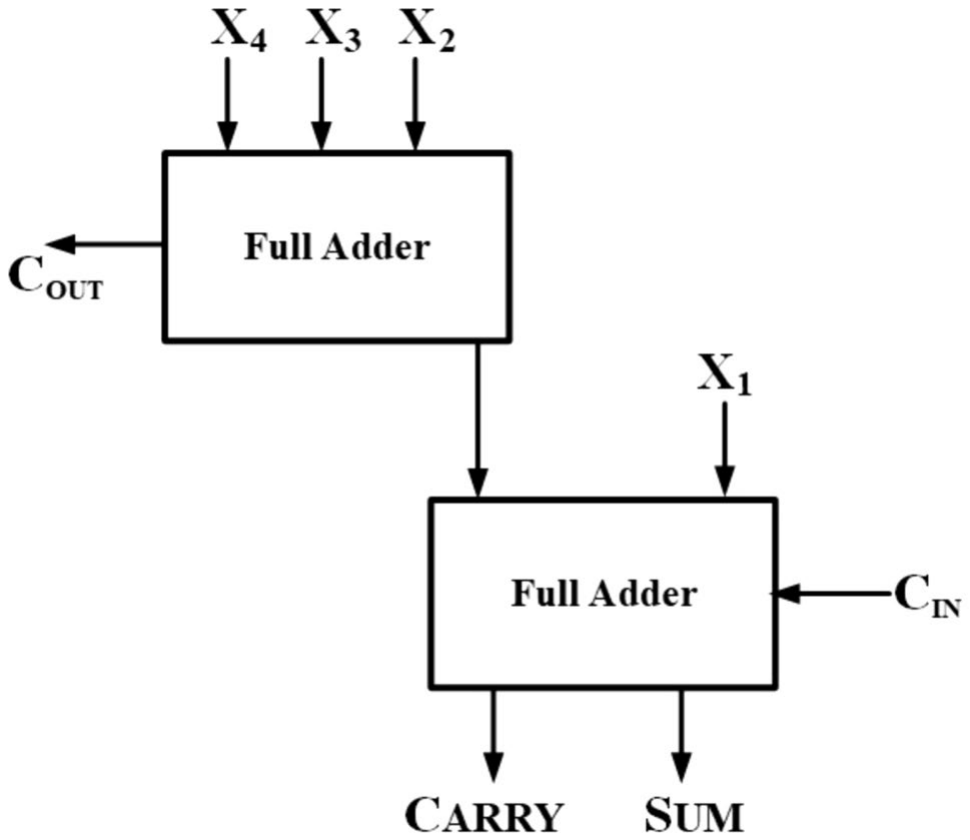
Internal architecture of 4:2 Exact compressor.

A 4:2 compressor comprises five inputs (X1, X2, X3, X4, $C_{in}$) and three outputs (Sum, Carry, $C_{out}$). The inputs and the Sum output share the same binary weighted value. The compressor takes the $C_{in}$ input from a preceding block with one less significant binary bit and yields $C_{out}$ and Carry outputs with one more significant binary bit. The system schematic of a precise 4:2 compressor is illustrated in Fig. 12, while its complete architecture is depicted in Fig. 13. The corresponding logical function of its respective outputs is shown in the below equations from (4), (5), (6):

4 $$\begin{aligned} Sum= & X_{1} \oplus X_{2} \oplus X_{3} \oplus X_{4} \end{aligned}$$

5 $$\begin{aligned} Carry= & \left( X_{1} \oplus X_{2} \oplus X_{3} \oplus X_{4} \right) C_{in} + \left( \overline{X_{1} \oplus X_{2} \oplus X_{3} \oplus X_{4}} \right) C_{in} \end{aligned}$$

6 $$\begin{aligned} C_{out}= & \left( X_{1} \oplus X_{2} \right) X_{3} + \left( \overline{X_{1} \oplus X_{2} } \right) X_{1} \end{aligned}$$

### Existing 4:2 inexact compressors

The article^7^ design employs two multiplexers to generate outputs based on the select line $X_{4}$, facilitating efficient parallel computation in multi-bit operations. The approximate compressor has a 12.5% error rate, indicating that 2 out of 16 input combinations yield incorrect outputs, making it more reliable than many other approximate compressors and full adder designs. It primarily utilizes two multiplexers and logic gates that implement the necessary Boolean equations, reducing output bits and simplifying subsequent compression stages in a multiplier. However, inaccuracies can be significant, especially in the most significant bit (MSB) columns, potentially impacting precision in critical applications.

In the article^12^, approximate 4:2 compressors, such as dual-quality (DQ) structures, streamline design to boost speed and lower power consumption at the expense of higher error rates. DQ4:$2C_{1}$ and DQ4:$2C_{2}$, for example, has a 62.5% error rate due to direct carry output approximation, while DQ4:$2C_{3}$ and DQ4:$2C_{4}$ enhance accuracy with additional gates, increasing complexity and power demands with an error rate of 50% and 31.2%. These designs improve efficiency but may not suit precision-critical applications due to accuracy trade-offs and integration challenges, necessitating careful selection based on application needs.

In the work 4:2, the compressor uses full-adder cells to condense four input bits into two output bits, enhancing multiplication efficiency. This research introduces two approximate 4:2 compressors, substituting traditional full adders with approximate versions for better performance. The first design drastically cuts transistor count, power consumption, and delay, though it has a 53% error rate, producing 17 incorrect results out of 32, making it unsuitable for precision-critical uses. The second design boosts accuracy but still carries the inherent imprecision of approximate computing. These compressors improve efficiency, but their reliability trade-offs must be considered in practical applications^13^.

A 4:2 compressor condenses four input bits into two output bits, managing carry-out for higher stages. Exact compressors use three XOR gates, one XNOR gate, and two 2:1 multiplexers for accurate arithmetic operations. Approximate compressors have a trade-off between accuracy and performance for compressors like Compressor3, Compressor4, and Compressor5, reducing errors to 3, 2, and 1, respectively. These designs boost power and area efficiency but introduce inaccuracies and integration challenges, making them less suitable for precision-critical applications. Still, it doesn’t integrate with an error compensation module to maintain a balancing precision with processing speed of the proposed compressor and the approximate multiplier^14^.

The approximate compressor in the article^15^ streamlines hardware by ignoring low-probability terms, resulting in a low error probability ($P_{E}$ = 13/256) . Utilizing only two AND-OR gates proves more efficient than designs needing extra gates like XOR and NOR, which raise error rates. However, its performance can suffer with uniformly distributed inputs, causing more errors. Although it improves hardware simplicity and power efficiency, its accuracy trade-offs must be carefully evaluated for precision-sensitive applications.

The 4:2 compressor design modifies the traditional truth table to control error rates while simplifying logic. It consistently produces outputs slightly below exact values, maintaining a predictable error profile easily corrected with a simple recovery module. Using fewer logic gates, primarily AND & OR gates, reduces hardware area and power consumption. A 2-level AND-OR circuit corrects errors at critical points, especially in the most significant bit. While it enhances efficiency by trading accuracy for hardware and power savings, it is less suitable for precision-critical applications. Integrating error recovery adds complexity, potentially offsetting some benefits^16^.

In the paper^24^, The 4:2 compressor design efficiently merges four input bits into two output bits with a 25% error rate and an error distance of ±1. Using 45nm CMOS technology, implemented logic gates and cascading compressors help balance error probabilities, enhancing performance. However, variability in error distance affects reliability, and design effectiveness varies by application. Reduced accuracy may be suitable for error-resilient contexts like image processing but not for critical systems. Additional error recovery mechanisms can complicate the design and offset benefits. Despite improvements, this design may lack the accuracy of architectures with dedicated error recovery modules that increase resource usage.

Y.Guo et al.^25^ implemented a 4:2 compressor use of AND & OR gates to eliminate the necessity for XOR gates, streamlining the circuit and lowering both area and power consumption in the multiplier, which was implemented in the proposed article on compressor for energy efficiency circuitry.

The PAC-1 and PAC-2 approximate 4:2 compressors use XOR and XNOR gates for compression, balancing accuracy and efficiency. PAC-1 has an error distance of ±2 with a 12.5% error rate, making it more reliable than PAC-2, which has an error distance of ±1 but a 50% error rate. While these designs lower area and power consumption, they compromise precision, making them unsuitable for high-precision or real-time applications. Additionally, managing component count and error rates adds complexity, posing challenges for practical implementation^26^.

### Proposed 4:2 inexact compressors

The following logic equations employed in the approximate 4:2 compressor design are shown in Eq. (7) with the related truth table presented in Table 2. This proposed design generates errors with an error rate of 26$\%$ and avoids using the XOR and XNOR gates in the logic equation to reduce the compressor’s power consumption and in the overall circuit. Moreover, the compressor is designed using only a 2,3 input NOR gate, which still reduces the circuit component and maintains a balance between performance and accuracy of the approximate multiplier, as indicated by the term differences in the truth table. Table 3 shows the Error Differences (ED) and Error Rates (ER), which maintain the minimum ER for producing improved accuracy metrics and reducing the error metrics.

The proposed 4:2 compressor demonstrates a favorable trade-off between approximation and precision by achieving a controlled error rate of 26$\%$ and a constrained error distance limited to -1 and -2. This behavior is more reliable than that of several prior designs, which tend to exhibit higher error rates and broader or more variable error distances. For example, DQ4:$2C_{1}$ and DQ4:$2C_{2}$^12^ incur error rates up to 62.5$\%$, and^13^ shows 56.6$\%$ with similarly loose error bounds. Other compressors, such as those in^16^, exhibit multiple error levels ranging from -1 to -4, leading to less predictable outcomes. In contrast, the error characteristics of our design remain consistent and bounded, offering improved predictability an essential requirement in low-power applications where maintaining acceptable accuracy is critical.

One of the central innovations of the work is the significant reduction in transistor usage, achieved without sacrificing circuit performance. The proposed 4:2 compressor is implemented using only 10 transistors, a notable improvement over prior designs such as^39^, each requiring 54 transistors. This reduction not only minimizes silicon footprint but also lowers static power dissipation and simplifies the manufacturing process. In comparison, designs reported in^13^ and^14^ often utilize between 25 and 80 transistors, highlighting the compactness advantage of our implementation as shown in Table 4. Beyond transistor-level efficiency, the gate-level structure of our design also promotes hardware simplicity. Unlike earlier compressors that rely heavily on XOR, XNOR, NAND, and AND logic–often resulting in increased power usage and propagation delay–our design is built using only two NOR gates. For instance, the compressor in^13^ includes a mix of AND, OR, NOR, and XNOR gates, and others such as^14^ and^28^ use diverse and complex logic gate arrangements across multiple stages as shown in Table 6. In contrast, our streamlined design reduces logical depth and wiring complexity, enhancing energy efficiency and making it more suitable for scalable and low-power applications.

**Figure 14:**
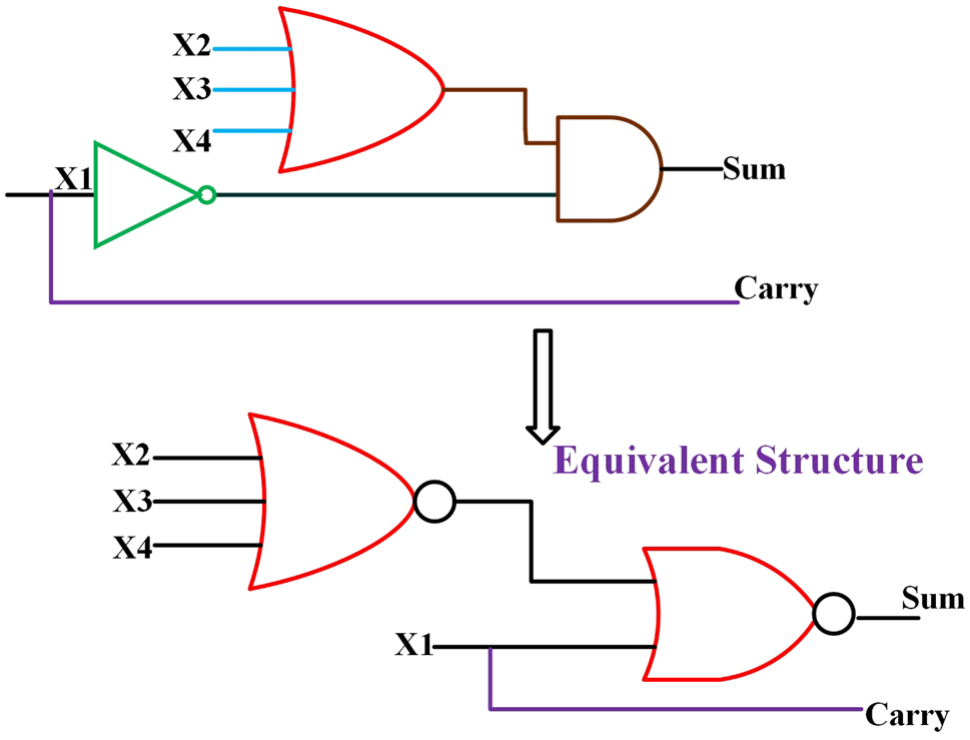
Proposed 4:2 Novel Compressor and its equivalent structure.

7 $$\begin{aligned} Sum = \left( X_{2} + X_{3} + X_{4} \right) \overline{X_{1}} ; Carry = X_{1} \end{aligned}$$

**Table 2:** Truth table of the proposed 4:2 compressor with error differences (ED).

$X_{4}$	$X_{3}$	$X_{2}$	$X_{1}$	Probability of occurrence	Carry	Sum	ED
0	0	0	0	81/256	0	0	0
0	0	0	1	27/256	1	0	$-1$
0	0	1	0	27/256	0	1	0
0	0	1	1	9/256	1	0	0
0	1	0	0	27/256	0	1	0
0	1	0	1	9/256	1	0	0
0	1	1	0	9/256	0	1	$-1$
0	1	1	1	3/256	1	0	$-1$
1	0	0	0	27/256	0	1	0
1	0	0	1	9/256	1	0	0
1	0	1	0	9/256	0	1	$-1$
1	0	1	1	3/256	1	0	$-1$
1	1	0	0	9/256	0	1	$-1$
1	1	0	1	3/256	1	0	$-1$
1	1	1	0	3/256	0	1	$-1$
1	1	1	1	1/256	1	0	$-2$

**Table 3:** Comparison of the proposed 4:2 compressor with error rates (ER) and ED to existing.

Reference	Error rate (ER)	ED
^7^	$12.5\%$	$\pm 1$
DQ4:$2C_{1}$^12^	$62.5\%$	$\pm 2$
DQ4:$2C_{2}$^12^	$62.5\%$	$\pm 2$
DQ4:$2C_{3}$^12^	$50\%$	$-2$
DQ4:$2C_{4}$^12^	$31.25\%$	$+2$
$De_{1}$^13^	$56.6\%$	$\pm 1$
$De_{2}$^13^	$39\%$	$\pm 1$
^14^	$18.75\%$	$-2$
^15^	$56.25\%$	$+1$
^16^	$25\%$	$-1,-2,-3,-4$
^18^	$68.3\%$	$\pm 1$
^24^	$25\%$	$\pm 1$
^26^	$39\%$	$\pm 1$
^27^	$53.1\%$	$\pm 1$
^28^	$29.6\%$	$\pm 1$
Proposed compressor	$26\%$	$-1,-2$

**Table 4:** Performance metrics of CMOS designs at 1 V compared with proposed NCFET-based compressor at 1 V.

References	Delay ($ps$)	Power ($\mu W$)	PDP ($aJ$)	EDP ($aJ\cdot ps$)	Number of Tx
^7^	48.8	3.54	172.75	8430	58
DQ4:$2C_{1}$^12^	12.2	0.86	10.49	127.9	4
DQ4:$2C_{2}$^12^	12.2	1.08	13.17	160.6	6
DQ4:$2C_{3}$^12^	41.5	2.63	109.14	4529.3	20
DQ4:$2C_{4}$^12^	4.3	3.41	14.66	63	30
$De_{1}$^13^	47.6	3.45	164.22	7816.8	28
$De_{2}$^13^	40.6	3.39	137.63	5587.7	26
^14^	61.84	3.97	245.5	15187.9	82
^15^	17.31	1.83	31.67	548.2	28
^16^	47.1	3.62	170.5	8030	76
^24^	25.48	2.48	63.19	1610	44
$De_{1}$^39^	38	0.295	11.21	425.98	54
$De_{2}$^39^	46	0.293	13.47	619.62	54
Proposed @ 1 V	10	0.269	2.69	26.9	10

$^{*}$ Only results from designs simulated at 45nm or properly scaled to this node are considered to ensure fair benchmarking. Performance metrics of designs proposed in^12,13,16^ are taken from^7^, and designs from^14,15^ are taken from^24^ implemented using 45 nm CMOS technology

**Table 5:** Normalized performance metrics of CMOS designs at 0.5 V compared with proposed NCFET-based compressor at 0.5 V.

References	Delay (ps)	Power ($\mu W$)	PDP (aJ)	EDP (aJ$\cdot$ps)
^7^	97.6	0.885	86.375	8430
DQ4:2C_1_^12^	24.4	0.215	5.245	127.9
DQ4:2C_2_^12^	24.4	0.27	6.585	160.6
DQ4:2C_3_^12^	83.0	0.6575	54.57	4529.3
DQ4:2C_4_^12^	8.6	0.8525	7.33	63
^13^De_1_	95.2	0.8625	82.11	7816.8
^13^De_2_	81.2	0.8475	68.815	5587.7
^14^	123.68	0.9925	122.75	15187.9
^15^	34.62	0.4575	15.835	548.2
^16^	94.2	0.905	85.25	8030
^24^	50.96	0.62	31.595	1610
^39^De_1_	76.0	0.07375	5.605	425.98
^39^De_2_	92.0	0.07325	6.735	619.62
**Proposed @ 0.5 V**	**4.0**	**0.03**	**0.12**	**0.48**

^*^ CMOS metrics normalized to 0.5 V assuming Delay $\propto$ 1/V, Power $\propto$ V^2^, PDP $\propto$ V, EDP constant

**Table 6:** Comparison of the proposed 4:2 compressor with different components to existing compressors.

Gates	ExoR		NAND		NOT	AND		**OR**		NOR		Ex-NOR
No. of inputs/reference	2	3	2	3	1	2	**3**	**2**	**3**	2	3	2
^7^	–	1	–	–	2	7	–	3	–	–	–	–
DQ4:$2C_{3}$^12^	–	–	1	–	–	–	–	–	–	–	–	2
DQ4:$2C_{4}$^12^	–	–	4	–	–	–	–	–	–	–	–	2
$De_{1}$^13^	–	–	–	–	–	–	–	–	–	5	–	2
$De_{2}$^13^	–	–	–	–	–	–	–	–	–	3	–	2
$De_{1}$^14^	–	–	–	–	6	4	2	4	1	–	–	–
$De_{2}$^14^	–	–	–	–	12	6	5	2	3	–	–	–
$De_{3}$^14^	–	–	–	–	11	5	5	3	1	–	–	–
^15^	–	–	–	–	–	2	–	–	2	–	–	–
^16^	2	–	–	–	–	5	–	3	1	–	–	–
^24^	1	–	–	–	1	3	–	3	–	–	–	–
^27^	–	–	–	–	–	2	–	2	–	–	–	–
^28^	–	–	–	–	2	6	–	2	1	–	–	–
$De_{1}$^39^	–	–	1	–	1	3	–	5	–	–	–	–
$De_{2}$^39^	–	–	–	–	1	4	–	4	–	1	–	–
Proposed compressor	–	–	–	–	–	–	–	–	–	1	1	–

**Figure 15:**
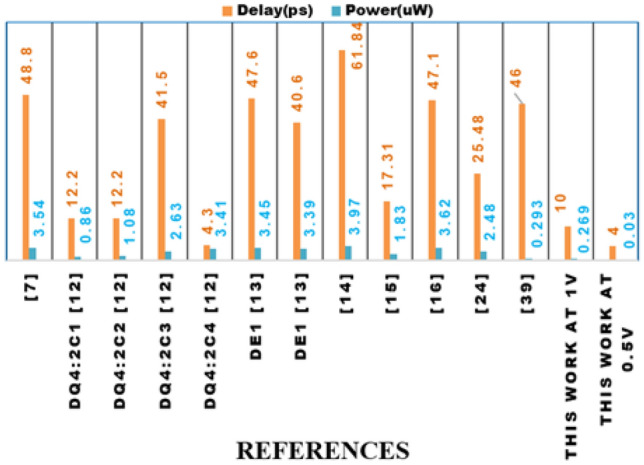
Performance comparison of the proposed 4:2 compressor with existing designs in terms of delay (ps) and power consumption ($\mu$W).

**Figure 16:**
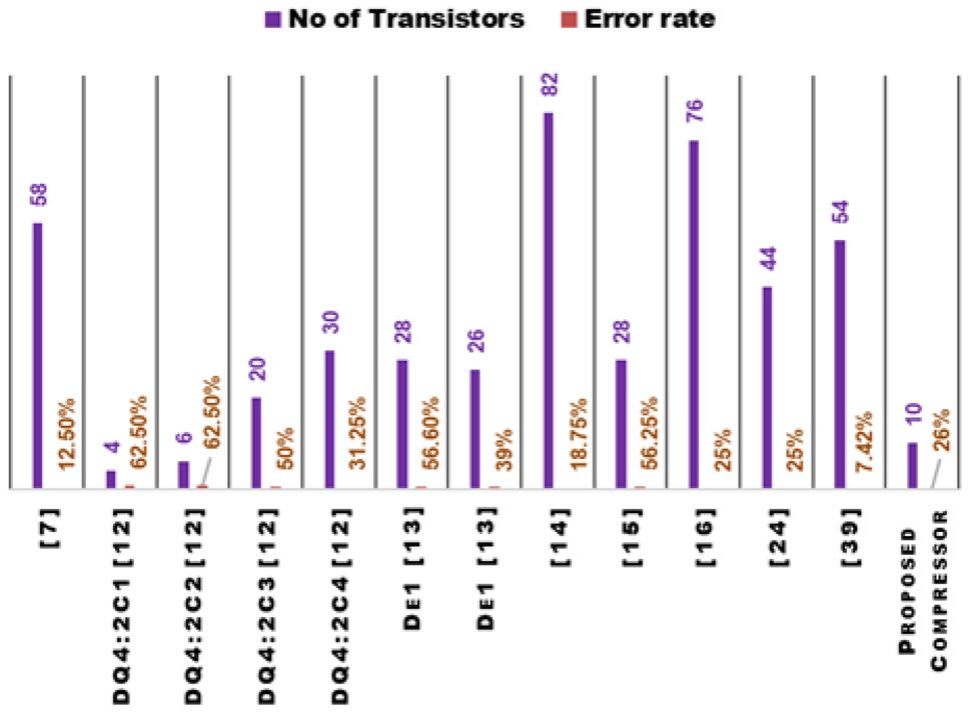
Performance comparison of the proposed 4:2 compressor with existing designs between no of transistors and error rate (ER).

**Figure 17:**
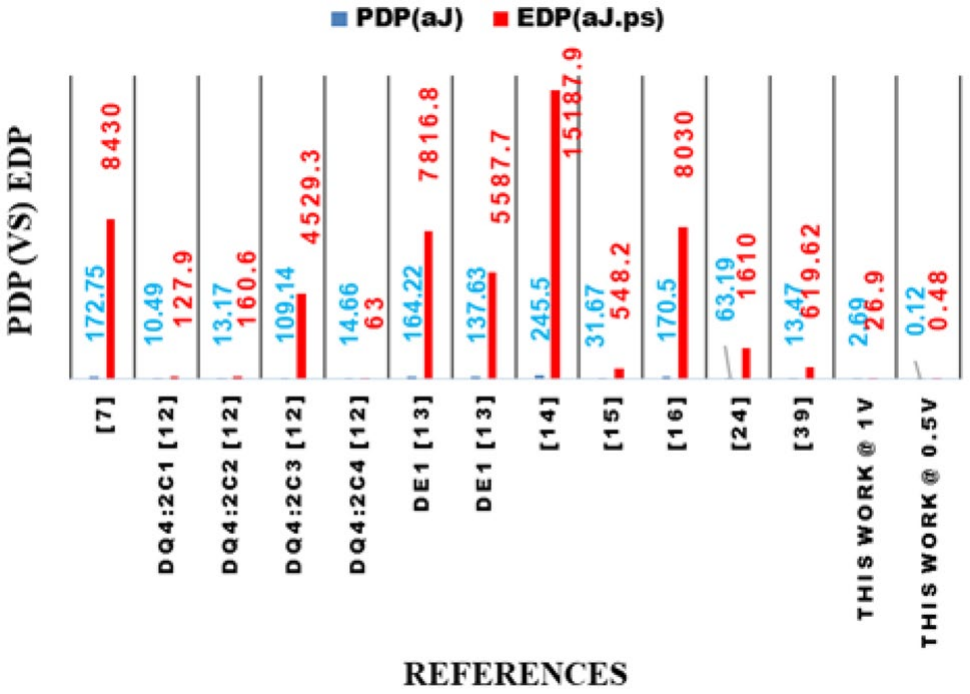
Performance comparison of the proposed 4:2 compressor with existing designs between PDP & EDP.

**Figure 18:**
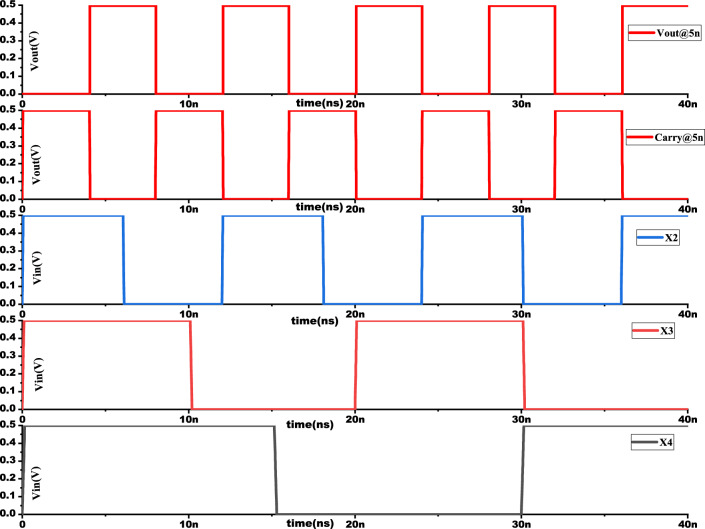
Output waveform of proposed novel 4:2 Compressor with $t_{fe}$ 5 nm.

For designing an 8*8 approximate multiplier, the proposed compressor excels with an error rate of 26% and error distances of − 1 and − 2, showcasing its superior performance compared to other references. This lower error rate and consistent error distances ensure high precision and minimal calculation variance, making it a reliable choice for efficient arithmetic operations. Conversely, references such as^12^ and^18^ display higher error rates (62.5% and 68.3%, respectively) and more significant variability in error distances, potentially leading to less accurate results and inefficiencies. Although some references, like^7^ and^13^, exhibit comparable error distances, their higher error rates highlight the advantage of the proposed compressor’s balance between precision and performance. Overall, the proposed compressor significantly improves error rate and stability, enhancing computational efficiency, reducing power consumption, and increasing processing speed, making it an ideal solution for applications that require reliable and precise approximate arithmetic operations.

The proposed 4:2 novel compressor is designed in cadence virtuoso with Verilog-A models from MVSNC source of NCFET device with a technology node of 45nm, and it is operated at 0.5V and 1V in room temperature at 1GHz frequency with a load capacitance of 1fF is shown in Fig. 14 which is more beneficial for low power consumption, and it performed well in hardware performance when compared to traditional CMOS designs, as more heat is dissipated from the very compact integrated chips in the present electronic world is a significant concern by using traditional CMOS. Table 4 and Table 5 shows the remarkable hardware performance of the proposed novel 4:2 compressor compared to existing compressors as same is represented in graphical represenation of hardware performance in Figs. 15, 16 and 17, as the proposed work also reduced the utilization of components, as shown in Table 4.

Figures 15, 16 and 17 present a detailed graphical analysis comparing the proposed 4:2 compressor with recent designs across several key performance parameters. In Fig. 15, the delay and power consumption trends clearly show that the proposed architecture offers reduced latency and energy usage relative to most prior designs, reflecting the efficiency gains achieved through NCFET technology. Figure 16 highlights the design’s compactness and accuracy, demonstrating that it uses only 10 transistors while maintaining a low error rate of 1.26%, outperforming many alternatives with significantly higher hardware complexity and less accuracy. Figure 17 underscores the energy advantages of our design by comparing the PDP and EDP. At 0.5V, the proposed compressor delivers a remarkably low PDP of 0.12aJ and EDP of 0.48aJ$\cdot$ps, surpassing all other referenced designs. Even at 1V, its energy metrics remain competitive. Together, these visual evaluations reinforce the proposed design’s strengths in terms of speed, energy performance, and circuit simplicity, making it a compelling option for low-power and approximate computing applications.

The output waveform of the proposed 4:2 novel compressor is shown in Fig. 18 which is simulated with different $t_{fe}$ at 5nm was guided by insights from our NCFET-based device simulations, specifically focusing on the $t_{fe}$ range of 3nm to 5nm. This selection is substantiated by the characteristics observed in Figs. 3 and 4, where increased ON-current values–rising to 129mA at 5nm from 105mA at 0nm indicate stronger drive capability. In addition, Fig. 9 demonstrates steeper voltage transfer curves and enhanced logic transition sharpness within this $t_{fe}$ range, suggesting improved switching behavior. Additionally, Fig. 6 demonstrates a significant decline in OFF-state current as $t_{fe}$ increases–from approximately 3$\mu$A at 0nm to just 10nA at 5nm–indicating improved leakage control and lower standby power consumption. Moreover, Fig. 7 identifies the 3-5nm range as an optimal design space that balances strong ON-current performance with minimal OFF-state leakage, making it highly suitable for energy-efficient and reliable logic circuit operation.To validate that the proposed 4:2 compressor operates reliably under these optimal device parameters, we simulated the output waveforms at these three $t_{fe}$ values. The resulting waveforms confirm stable functionality without delay variation or signal degradation. Nonetheless, we appreciate the reviewer’s recommendation and are open to retaining only one representative waveform $t_{fe}= 5nm$ to maintain focus and clarity in the revised manuscript.

The proposed 4:2 compressor demonstrates leading edge performance across key metrics, including delay, power, and energy efficiency. At a supply voltage of 1V, the design achieves a delay of just 10ps, consumes 0.269$\mu$W of power, and exhibits a PDP of 2.69aJ. These figures further improve under low voltage operation at 0.5V enabled by the properties of NCFET devices with delay reducing to 4ps, power to 30nW, and PDP to 0.12aJ. Compared to recent designs such as^39^, which reports a 38ps delay, 295nW power consumption, and a PDP of 11.21aJ using significantly more transistors, our design offers substantial improvements. These gains are attributed not only to an efficient circuit architecture but also to the benefits of NCFET technology, including its sharp subthreshold swing and reduced leakage characteristics

For designing an 8*8 approximate multiplier, the proposed compressor with the NCFET Verilog-A model shows remarkable performance enhancements. It boasts an impressive delay reduction of up to 83.83% and a power consumption reduction of up to 93.23%. The PDP is improved by up to 98.9%, and the EDP sees an enhancement of up to 99.82% compared to other references. Furthermore, the proposed compressor significantly decreases the required transistors, with reductions ranging from 64.29% to 87.8%. These improvements underscore the proposed compressor’s superior performance, making it a highly efficient and reliable option for applications that demand precise and effective approximate arithmetic operations.

overall,A comprehensive statistical evaluation of the proposed 4:2 compressor against contemporary designs demonstrates its significant advantages across critical performance metrics, including delay, power, PDP, EDP, and transistor count. Operating at 1V, our design achieves a delay of just 10ps offering speed improvements ranging from 1.2$\times$ to over 6$\times$ compared to high-performing alternatives such as 17.31ps^15^ and 17.21ps^27^, and far outperforming slower counterparts like 48.8ps^7^ ,61.84ps^14^, and 47.1ps^16^. In terms of power consumption, the proposed design operates at just 0.269$\mu$W, demonstrating significant reductions compared to designs such as 3.39-3.45$\mu$W^13^, 3.54$\mu$W^7^, and 3.97$\mu$W^14^, while also maintaining an edge over more compact designs from^12^ like DQ4:$2C_{1}$ and $2C_{2}$ 0.86$\mu$W and 1.08$\mu$W, respectively. The PDP is notably efficient at 2.69aJ, which is 4$\times$ to nearly 90$\times$ lower than values reported in prior works, ranging from 10.49aJ in^12^ to as high as 245.5aJ in^14^. Additionally, the EDP stands at just 26.9aJ$\cdot$ps, offering a substantial advantage over figures such as 127.9 in^12^, 548.2 in^15^, and more than 15,000aJ$\cdot$ps in^14^. From a hardware perspective, the proposed compressor is extremely compact, utilizing only 10 transistors. This is markedly fewer than the 26-82 transistors used in designs like^7,13,14,16^, highlighting a 2.6 $\times$ to 8 $\times$ reduction in transistor count. Even when compared to the minimalist designs in^12^, which use 4-6 transistors, our design maintains competitive area efficiency while delivering superior energy metrics. Furthermore, the benefits of operating with NCFET technology become clear at reduced voltages: at 0.5V, the delay improves to 4ps, power drops to 30nW, PDP is reduced to 0.12aJ, and EDP to 0.48aJ$\cdot$ps–outperforming all CMOS-based counterparts across the board. These detailed comparisons underline the proposed design’s clear edge in performance, energy efficiency, and hardware simplicity.

Overall, it is observed from Fig. 18 that the performance of the proposed compressor remains robust across different $t_{fe}$ sizes, demonstrating its efficiency and resilience in various technology nodes. This consistency suggests that the NCFET Verilog-A model is well-suited for scaling to smaller feature sizes while maintaining reliable signal integrity and performance.

## Proposed 8*8 approximate multiplier

The proposed 8*8 approximate multiplier design is implemented using a novel 4:2 compressor comprising three stages. Stage 1 is segregated into three parts. For an 8*8 multiplier, there are 16 outputs named $Y_{0}$-$Y_{15}$, with $Y_{0}$-$Y_{3}$ being the truncated region. $Y_{4}$-$Y_{7}$ is the approximate region where we use the proposed approximate compressor and half adder.

**Figure 19:**
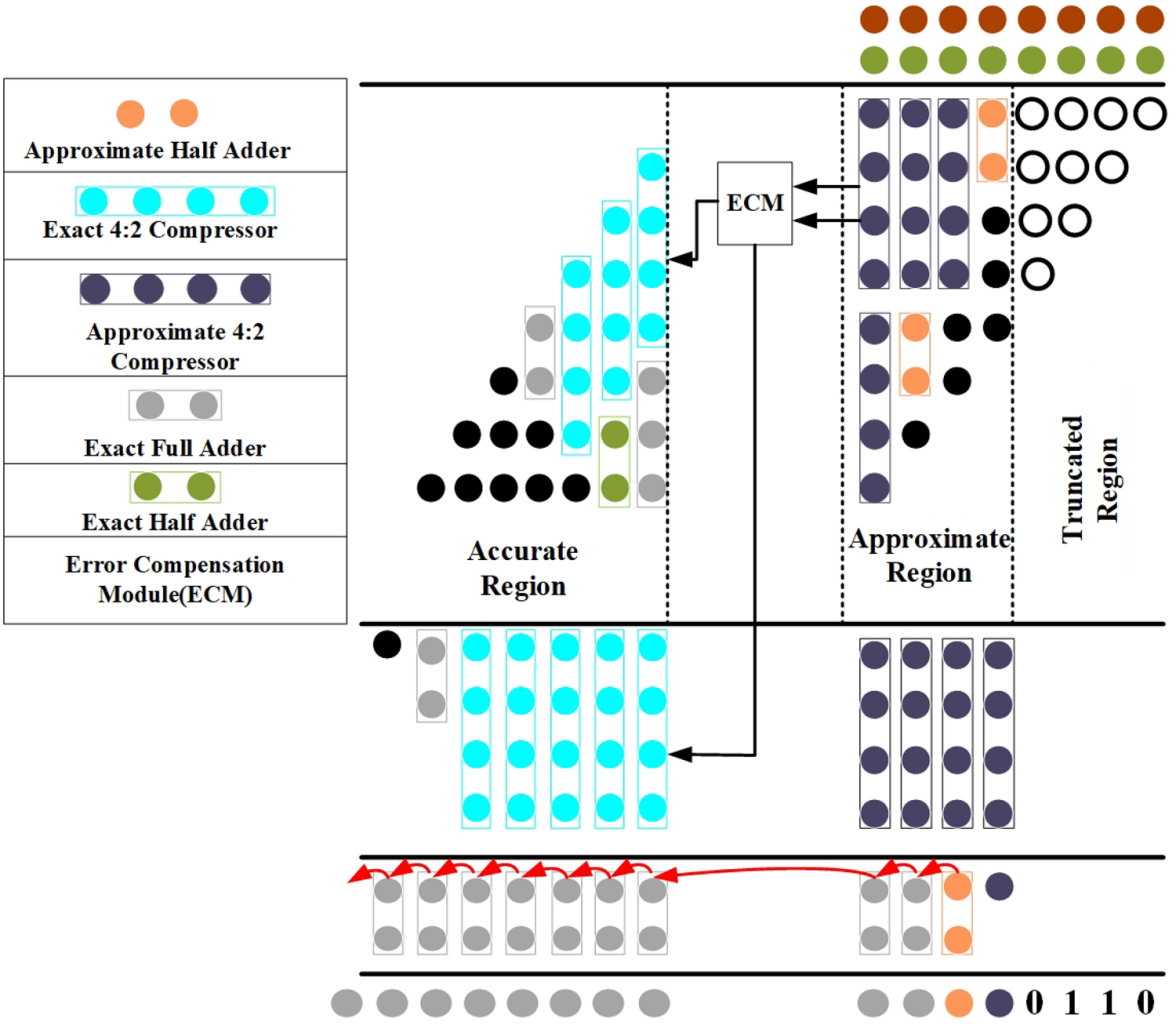
Proposed 8*8 approximate multiplier Architecture using proposed novel 4:2 Compressor.

**Table 7:** Performance metrics of 8*8 approximate multipliers at 1 V, including the proposed NCFET-based design.

Ref	Delay ($ns$)	Power ($\mu W$)	PDP ($fJ$)	EDP ($fJ\cdot ns$)
$De_{1}$^26^	0.34	50.63	17.21	5.85
$De_{2}$^26^	0.31	53.63	16.66	5.16
$De_{1}$^13^	0.6	217	130	78
$De_{2}$^13^	0.57	197	112	64
DQ4:$2C_{1}$^12^	0.35	98	34	12
DQ4:$2C_{2}$^12^	0.46	93	43	20
DQ4:$2C_{3}$^12^	0.49	194	95	46
DQ4:$2C_{4}$^12^	0.53	205	108	57
DQ4:$2C_{5}$^12^	0.52	135	70	37
^16^	0.50	79.24	39.62	20.6
^25^	0.48	74.33	35.48	17.03
^14^	0.49	80.19	39.29	19.255
^15^	0.31	39.22	12.16	3.76
^24^	0.33	51.01	16.83	5.55
^7^	0.357	56.52	20.18	7.2
**Proposed (NCFET, 1V)**	**0.74**	**42.2**	**31.2**	**23.108**
**Proposed (NCFET, 0.5V)**	**0.56**	**12.8**	**7.168**	**4.104**

^*^ Only results from designs simulated at 45nm or properly scaled to this node are considered to ensure fair benchmarking. Performance metrics of designs proposed in^13^ are taken from^12^,and designs from^14–16^ are taken from^24^ and designs from^25^ are taken from^26^ implemented using 45 nm CMOS technology

**Table 8:** Normalized performance metrics of 8*8 approximate multipliers at 0.5 V, including the proposed NCFET-based design.

Ref	Delay (ns)	Power ($\mu$W)	PDP (fJ)	EDP (fJ$\cdot$ns)
$De_{1}$^26^	0.68	12.66	8.605	5.85
$De_{2}$^26^	0.62	13.41	8.33	5.16
$De_{1}$^13^	1.20	54.25	65.00	78.00
$De_{2}$^13^	1.14	49.25	56.00	64.00
DQ4:$2C_{1}$^12^	0.70	24.50	17.00	12.00
DQ4:$2C_{2}$^12^	0.92	23.25	21.50	20.00
DQ4:$2C_{3}$^12^	0.98	48.50	47.50	46.00
DQ4:$2C_{4}$^12^	1.06	51.25	54.00	57.00
DQ4:$2C_{5}$^12^	1.04	33.75	35.00	37.00
^16^	1.00	19.81	19.81	20.60
^25^	0.96	18.58	17.74	17.03
^14^	0.98	20.05	19.65	19.26
^15^	0.62	9.81	6.08	3.76
^24^	0.66	12.75	8.42	5.55
^7^	0.714	14.13	10.09	7.20
**Proposed NCFET, 0.5V**	**0.56**	**12.8**	**7.168**	**4.104**

^*^ CMOS designs are normalized to 0.5 V using $\text {Delay}_{0.5\text {V}} \approx 2\times$, $\text {Power}_{0.5\text {V}} \approx 0.25\times$. EDP remains constant. All designs are benchmarked at 45 nm for fairness. Proposed NCFET-based multiplier is directly simulated at 0.5 V

**Table 9:** Comparison of the proposed 8*8 approximate multiplier with accuracy metrics to existing.

References	PDP (fJ)	EDP	MED	MRED	NMED	PDP* MRED	1-NMED
$De_{1}$^26^	17.21	5.85	162.76	0.0521	0.0025	0.8966	0.9975
$De_{2}$^26^	16.66	5.16	141.79	0.0699	0.0022	0.9996	0.9978
$De_{1}$^13^	130	78	3873.4	4.5483	0.0596	590.2	0.9404
$De_{2}$^13^	112	64	3508.6	4.2843	0.0546	479.36	0.9454
DQ4:$2C_{1}$^12^	34	12	3540	0.3692	0.0545	12.55	0.9455
DQ4:$2C_{2}$^12^	43	20	2400	0.2932	0.037	12.6	0.963
DQ4:$2C_{3}$^12^	95	46	3220	0.3281	0.0496	31.16	0.9504
DQ4:$2C_{4}$^12^	108	57	1390	0.0809	0.0213	8.73	0.9787
DQ4:$2C_{5}$^12^	70	37	1460	0.1194	0.0225	8.358	0.9775
$De_{1}$^16^	39.62	20.6	25.212	0.0326	0.0023	1.3	0.9977
$De_{1}$^16^	444.42	1200	25.212	0.0326	0.0023	14.48	0.9977
^25^	35.48	17.03	635.27	0.0292	0.0114	1	0.9886
^14^	39.29	19.25	6476	1.2	0.0537	47.1	0.9463
^15^	12.16	3.76	2888	0.3002	0.0444	3.65	0.9556
^24^	16.83	5.55	573.4	0.0487	0.0027	0.8196	0.9973
^7^	20.18	7.2	573.3	0.0895	0.0098	16.35	0.9902
**Proposed NCFET, 0.5V**	7.168	4.104	2870	0.0304	0.0409	0.217	0.9591
**Proposed NCFET, 1V**	31.228	23.108	2870	0.0304	0.0409	0.949	0.9591

**Figure 20:**
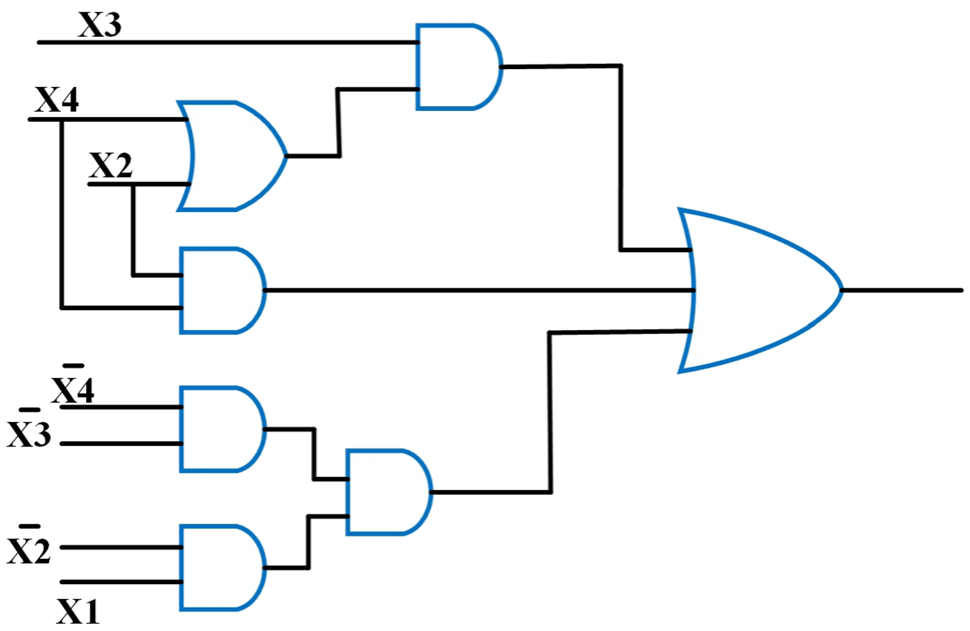
Error compensation module.

**Figure 21:**
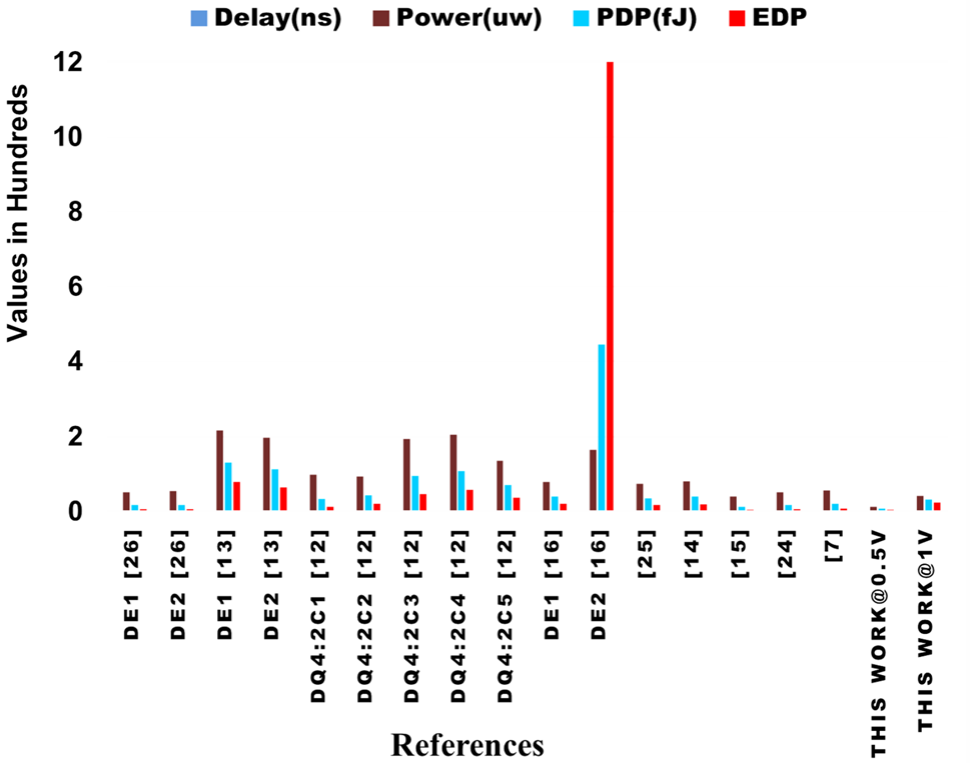
Graphical representation of proposed 8*8 approximate multiplier with existing w.r.to performance metrics.

**Figure 22:**
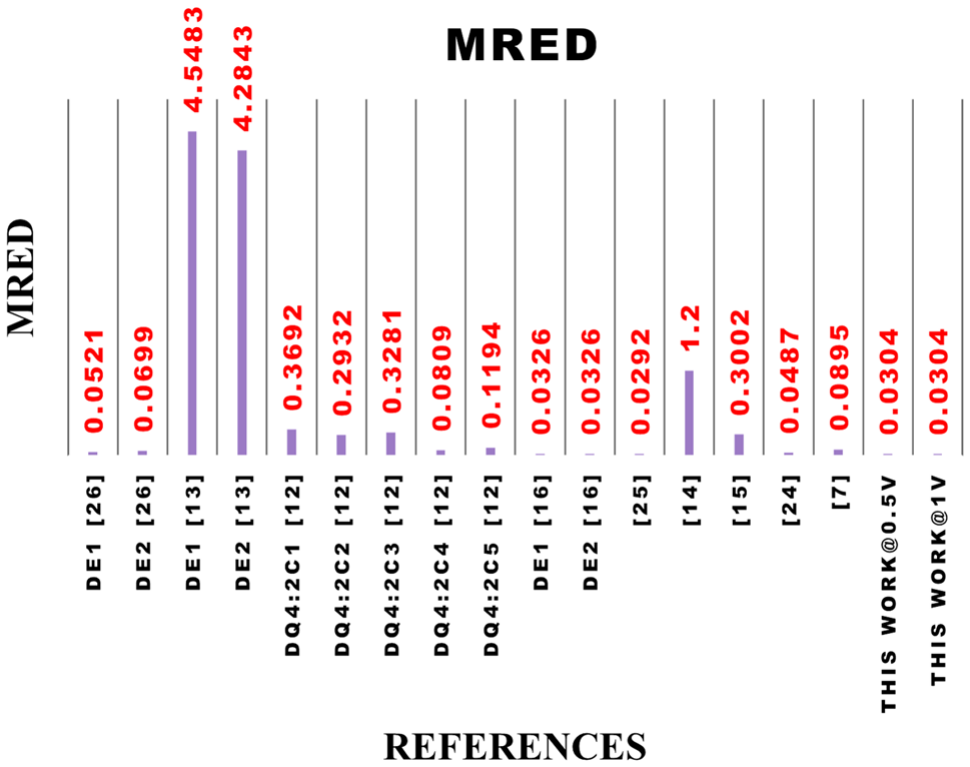
Graphical representation of proposed 8*8 approximate multiplier with existing w.r.to MRED.

**Figure 23:**
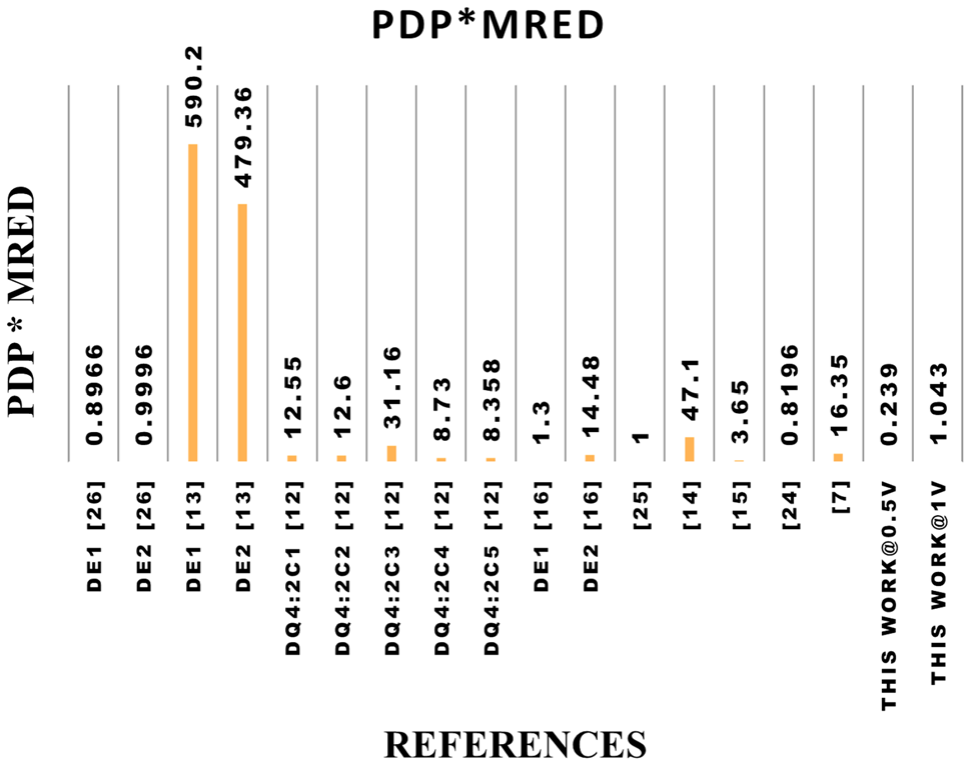
Graphical representation of proposed 8*8 approximate multiplier with existing w.r.to PDP*MRED.

**Figure 24:**
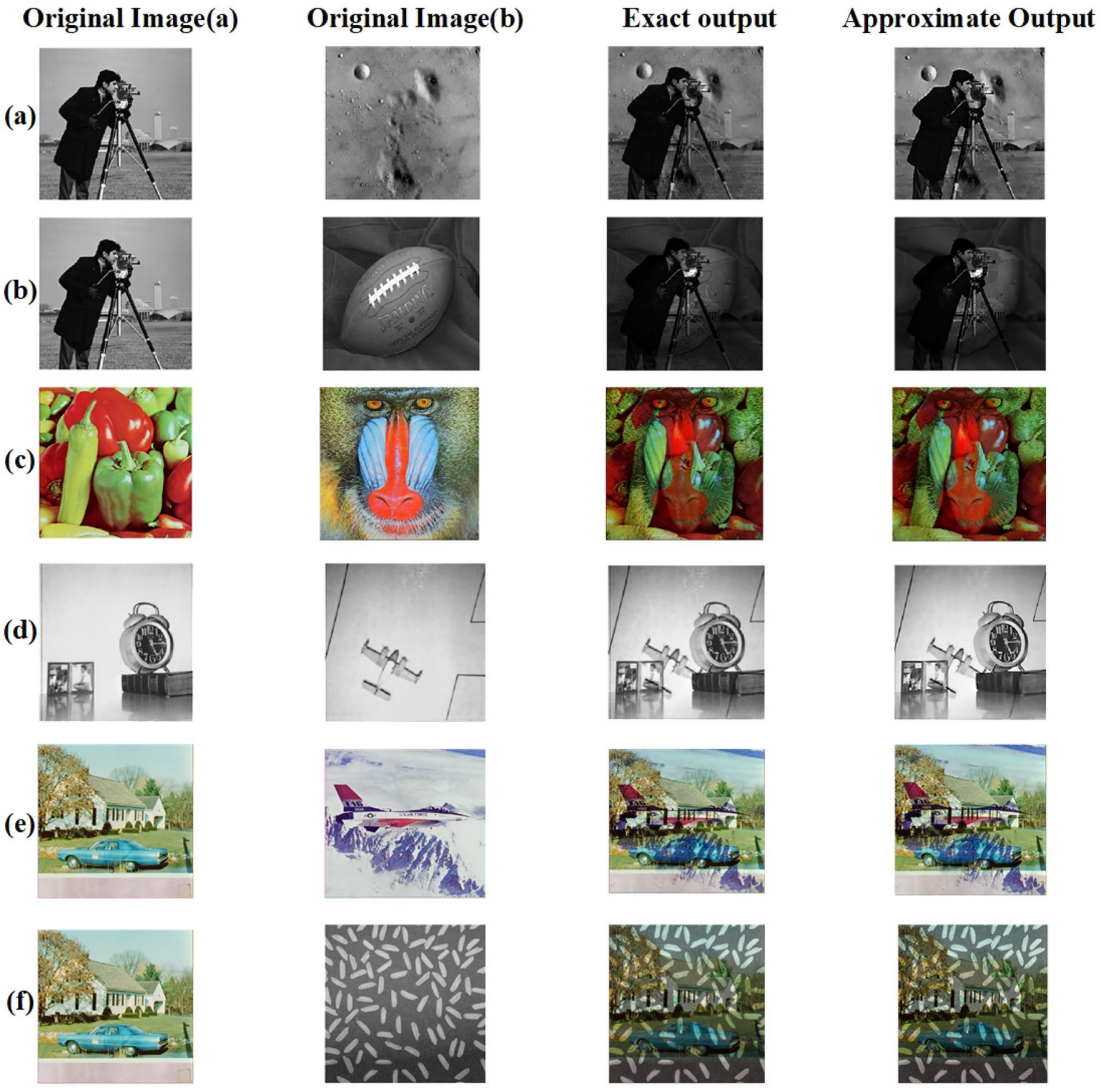
(**a**) Cameraman * Moon Surface, (**b**) Cameraman * Football, (**c**) Pepper * Mandrill, (**d**) Clock * airplane, (**e**) house * Jet, (**f**) house * Rice.

**Figure 25:**
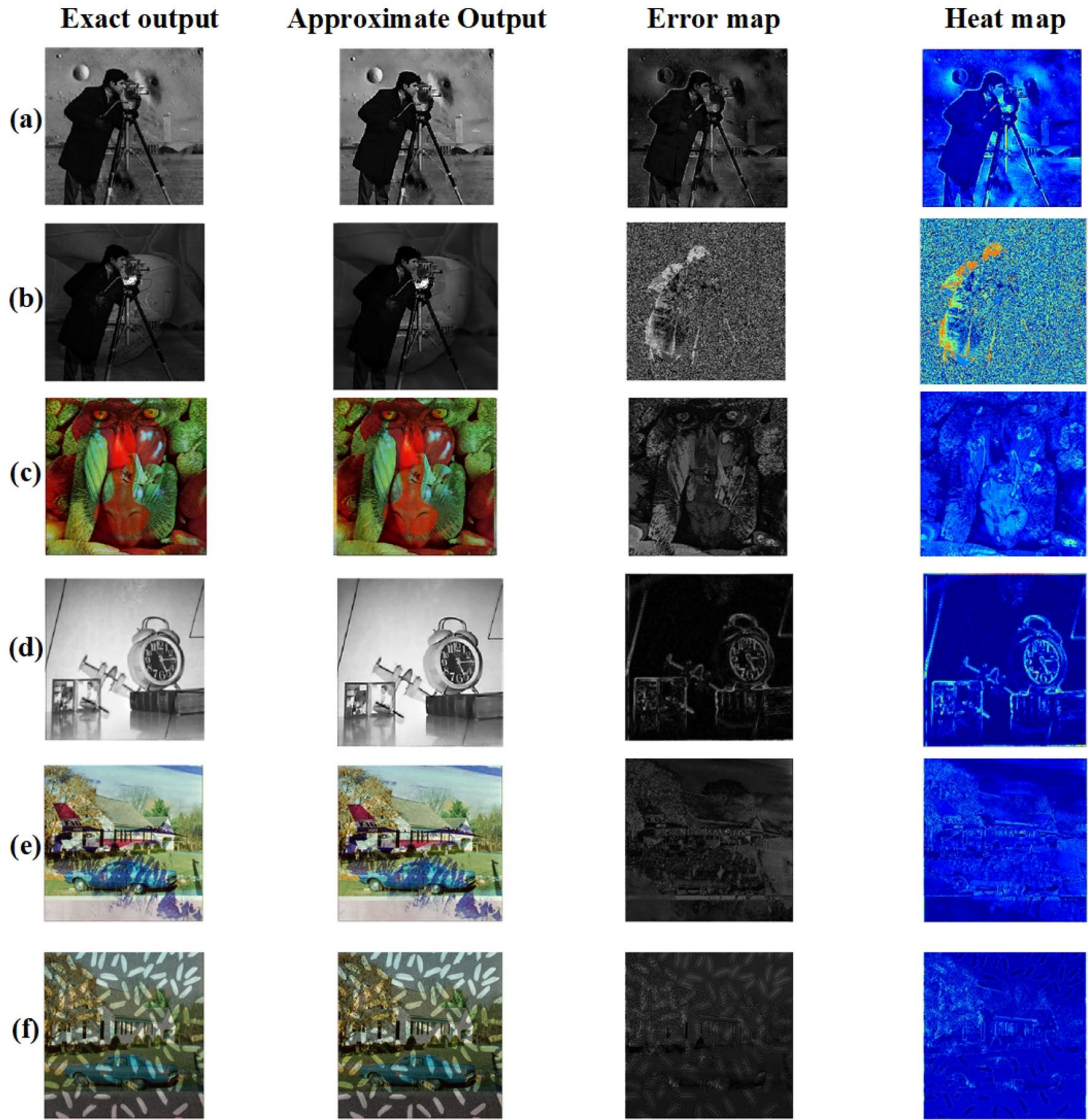
(**a**) Cameraman * Moon Surface, (**b**) Cameraman * Football, (**c**) Pepper * Mandrill, (**d**) Clock * airplane, (**e**) house * Jet, (**f**) House * Rice.

**Figure 26:**
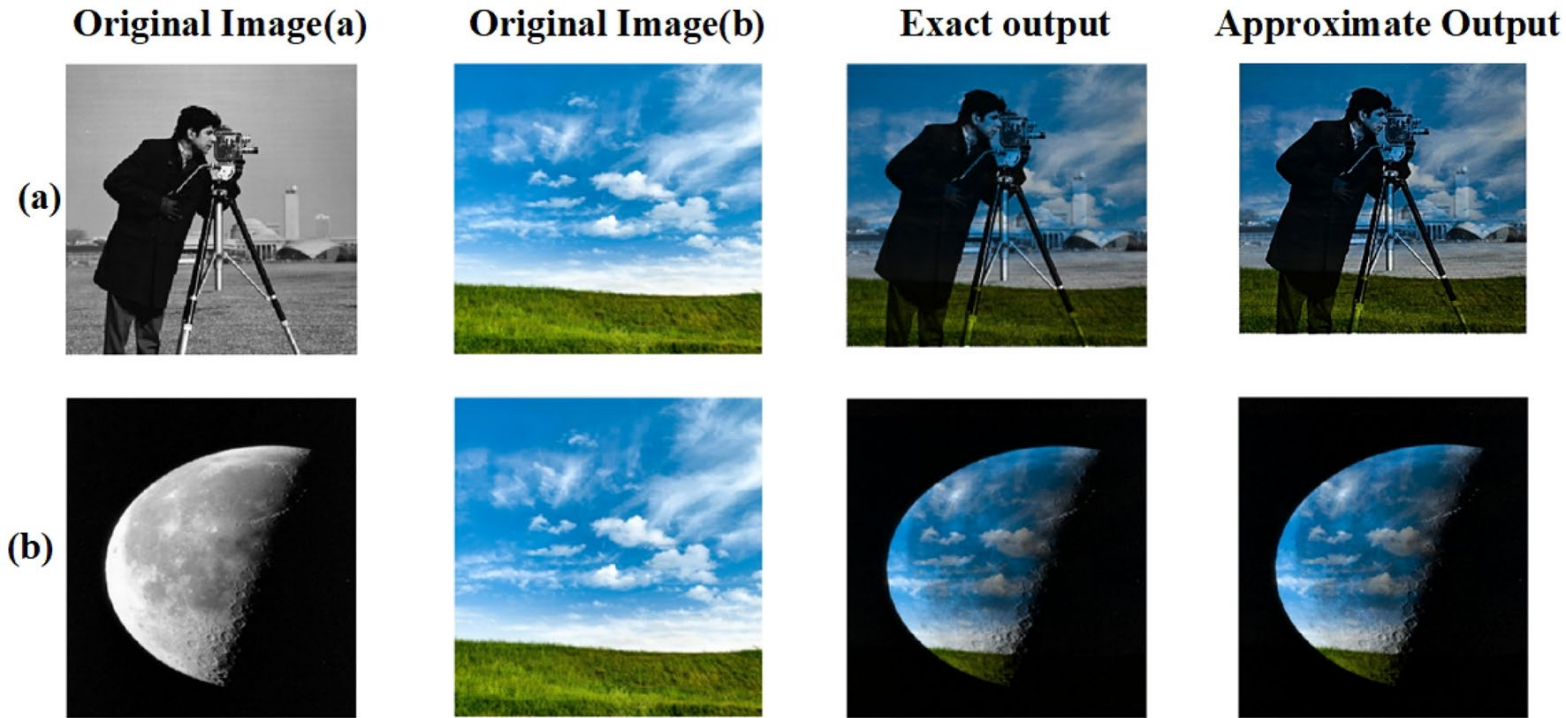
(**a**) Cameraman * Sky, (**b**) Moon * Sky.

**Figure 27:**
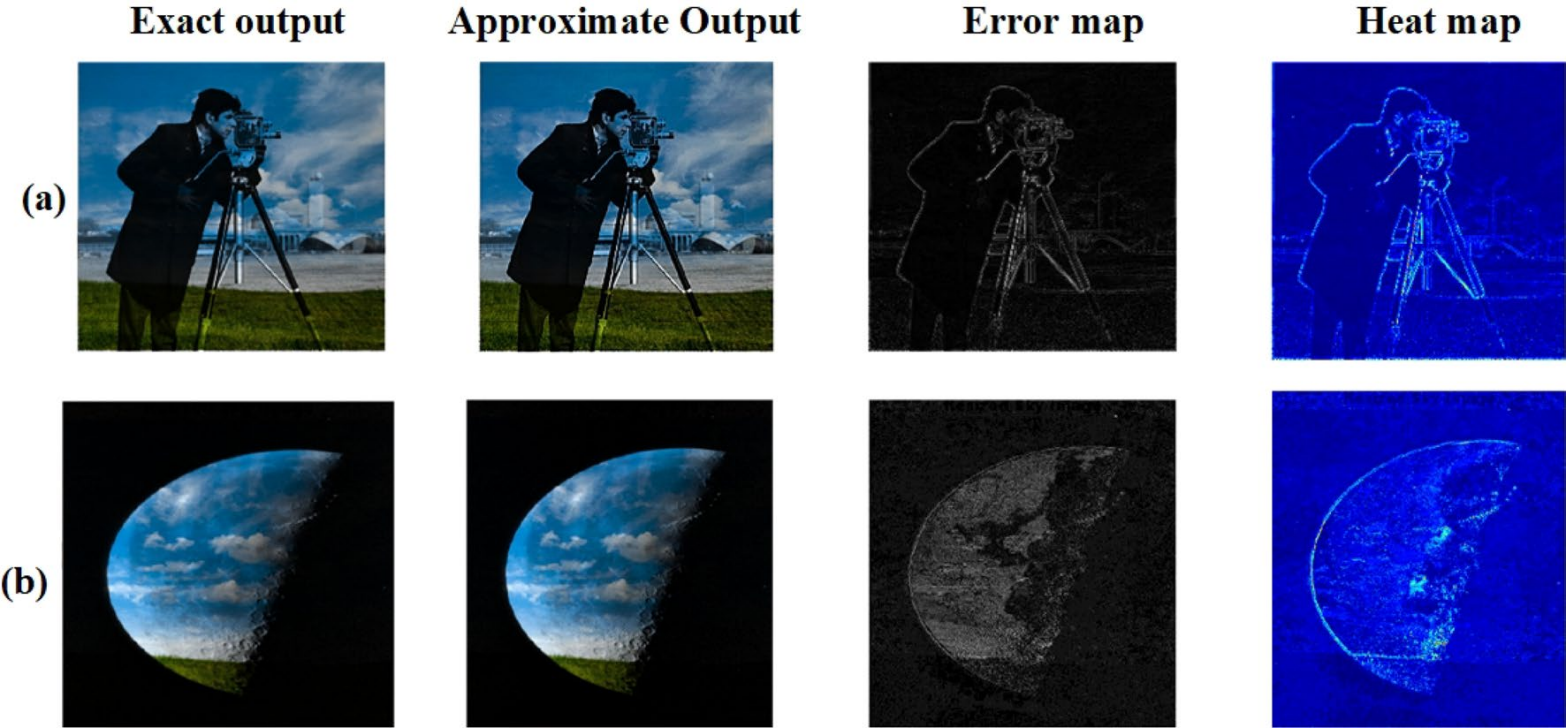
(**a**) Cameraman * Sky, (**b**) Moon * Sky.

**Table 10:** THE PSNR AND MSSIM of the proposed approximate multiplier.

S. no	Multiplication images	PSNR (dB)	MSSIM
1	Cameraman * Moon Surface	55.8	0.9469
2	Cameraman * Football	53.5	0.9685
3	Pepper * Mandrill	49.4	0.9264
4	Clock * airplane	41.5	0.9767
5	house * Jet	43.7	0.987
6	house * Rice	41.3	0.99
7	Cameraman * Sky	35.47	0.9875
8	Moon * Sky	45.36	0.98

**Table 11:** Mean SSIM analysis of standard images.

S. no	Cameraman * Moon surface	Clock * airplane
$De_{1}$ ^13^	0.65	0.6642
$De_{2}$ ^13^	0.68	0.6971
DQ4:$2C_{1}$^12^	0.60	0.6153
DQ4:$2C_{2}$^12^	0.65	0.6653
DQ4:$2C_{3}$^12^	0.64	0.6580
DQ4:$2C_{4}$^12^	0.81	0.83
^16^	0.93	0.952
^14^	0.86	0.8824
^15^	0.63	0643
^24^	0.90	–
^7^	0.92	0.943
Proposed work	0.9469	0.9767

**Table 12:** PSNR and MSSIM for image multiplications using different multipliers.

Multiplier	Sky $\times$ Moon		Sky $\times$ Cameraman	
	PSNR (dB)	MSSIM	PSNR (dB)	MSSIM
Proposed Mul	45.35	0.98	35.47	0.9875
Mul_1^13^	23.99	0.842	22.77	0.756
Mul_1^14^	37.34	0.989	37.24	0.987
Mul_2^14^	32.92	0.98	31.61	0.978
Mul_1^12^	31.58	0.974	30.58	0.977
Mul_1^41^	31.62	0.974	30.68	0.977
Mul_1^42^	31.91	0.971	30.56	0.965
Mul_1^43^	33.47	0.982	31.35	0.981
Mul_2^43^	33.72	0.983	32.36	0.981

The rest of the output, i.e. $Y_{8}$-$Y_{15}$, is produced using the exact compressor, full adder, and half adder. In general, for an m*m multiplier, the m/2 columns at the rightmost end of the partial product generated by the multiplier will get truncated according to the requirement, which is implemented in stage 1 for the proposed one, as shown in Fig.19.By truncating four columns as we are using 8*8 multiplier 10 partial products are truncated which reduce in hardware of 60 transistors in overall product output and which impact in improved efficiency and area of circuit. The multiplier is implemented in 45nm NCFET using cadence virtuoso operated at 0.5V and 1V, and for a fair comparison, it is compared with the existing multipliers of 45nm CMOS technology.

### Error compensation module

In Fig.19, the error recovery bit is produced explicitly in the most significant bit within the approximate region using an error compensation circuit. This placement is intended to maximize accuracy while keeping the hardware overhead minimal, and it is crucial to compensate for the errors of all compressors in this column.Fig.20.Error detection and correction are implemented as outlined in^16^. The detection mechanism focuses on the left-most approximate compressors, where error signals are generated. These signals are utilized to produce a $C_{in}$ input for the exact 4-2 compressor, which is positioned in the subsequent column of the partial product matrix. This process helps in reducing the multiplier error through error recovery. Importantly, the hardware resources required for error correction are minimal, as the recovery operation is confined to the most significant column within the approximate region. Additionally, the delay introduced remains negligible, thanks to the efficient use of the fast $C_{in}$ input in the exact 4-2 compressor as shown in Fig. 13.

### Hardware analysis

The multiplier is implemented in 45nm NCFET using cadence virtuoso operated at 0.5V and 1V, and for a fair comparison at operating frequency of 1GHz in a room temperature with a load capacitance of 1fF-2fF, it is compared with the existing multipliers of 45nm CMOS technology. The corresponding values are shown in Tables 7, 8 and Fig. 21. At 0.5V, the proposed compressor exhibits a delay of 0.56 ns, a power consumption of 12.8 $\mu$W, and a PDP of 7.168 fJ. When compared to references^15,24,26^, which have shorter delays by 36.3% to 44.6%, the power usage of these references is substantially higher, ranging from 206% to 319%, and their PDP values exceed the proposed compressor by 69.6% to 140%. Additionally, references^7,12–14,16,25^ show significantly higher power consumption, varying from 626% to 1595%, and their PDP values are 374% to 6103% greater. Despite having a marginally longer delay, the proposed compressor at 0.5V excels in lower power consumption and PDP, making it ideal for power-sensitive tasks such as an 8*8 approximate multiplier.

The 8*8 approximate multiplier, developed using the proposed 4:2 compressor, delivers exceptional results in terms of energy and speed. Operating at 0.5V, it achieves a delay of 0.56ns, power usage of 12.8$\mu$W, and yields a PDP of 7.168fJ and EDP of 4.104fJ$\cdot$ns. These figures are notably competitive when compared to recent architectures. For example, the DQ4:$2C_{1}$ design^12^ reports a PDP of 34fJ and an EDP of 12fJ$\cdot$ns, while consuming more power and employing deeper logic structures. Additionally, the proposed multiplier exhibits strong scalability across supply voltages, maintaining energy efficiency and consistent behavior. This makes it a robust choice for applications ranging from low-power portable systems to high-performance approximate computing tasks

The 8*8 approximate multiplier at 1V demonstrates a delay of 0.74 ns, power consumption of 42.2 $\mu$W, and a PDP of 31.228 fJ. In comparison, references^15,24,26^ exhibit delays that are 38.5% to 58.1% shorter. Despite this, the proposed compressor’s power consumption is only slightly higher, ranging from 7.1% to 20.5% above the reference^15^, while still being more efficient than other references by 21.8% to 65%. Moreover, the proposed compressor’s PDP is significantly better, with values 315% to 1440% lower than those of references^7,12–14,16,25^. Thus, although the delay at 1V is longer, the proposed compressor offers an advantageous balance of delay, power consumption, and PDP compared to the existing references. At a supply voltage of 1V, the proposed 8*8 approximate multiplier demonstrates a strong trade-off between performance and energy efficiency. While its delay of 0.74ns is slightly higher than that of the fastest designs–such as^15^ and^26^, both around 0.31–0.34ns, and^24^ at 0.33ns it remains well within acceptable limits for low-power applications. Importantly, the power requirement is considerably lower than that of many existing designs. For instance, designs like^13^ (197–217 $\mu$W),^12^ (93–205$\mu$W), and^14^ (80.19$\mu$W) exhibit substantially higher consumption. Even compared to optimized designs such as^25^ (74.33$\mu$W) and^7^ (56.52$\mu$W), the proposed design’s 42.2$\mu$W remains notably efficient. The PDP is also favorable at 31.2fJ, outperforming a majority of the existing multipliers including^12,13^, and^14^, while achieving performance close to the best-performing designs like^15^ (12.16fJ) and^24^ (16.83fJ). Additionally, the EDP of 23.108fJ$\cdot$ns reflects superior efficiency compared to alternatives such as^13^ (64–78fJ$\cdot$ns) and^12^ (up to 57fJ$\cdot$ns). These results validate the effectiveness of the proposed NCFET-based multiplier in delivering energy-efficient performance, even under nominal operating conditions, and make it a compelling candidate for energy-aware digital systems

## Accuracy analysis of the proposed design

### Error metrics

In this section, the accuracy factors of the evaluated multipliers are analyzed based on the results obtained through MATLAB simulations. To determine output quality, several additional illustrative metrics are considered beyond the error distance parameter. These include error rate (ER), error distance(ED), mean error distance (MED), mean relative error distance (MRED), and normalized mean error distance (NMED). These metrics comprehensively assess the output quality for approximate circuits in error-resilient applications. The disparity between imprecise outputs and the precise outputs of an approximate adder is known as the error distance (ED) as shown in Eq. (8). The definition of this parameter is as follows:

8 $$\begin{aligned} ED = \left| precise output_i- approximate output_i \right| \end{aligned}$$

An approximate multiplier’s ER is the likelihood of encountering a nonzero ED, meaning the count of incorrect outcomes for various input combinations as shown in Eq. (9). It is determined as follows:

9 $$\begin{aligned} ER= & \frac{\left( ED \ne 0 \right) }{\left( ED \ne 0 \right) + \left( ED = 0 \right) } \end{aligned}$$

10 $$\begin{aligned} MED= & \frac{1}{2^{2M}}\sum _{i=1}^{2^{2M}}\left| ED_{i} \right| \end{aligned}$$

Further, MED is defined by^29^ as represent in Eq. (10)

11 $$\begin{aligned} MRED= & \frac{1}{2^{2M}}\sum _{i=1}^{2^{2M}}\frac{\left| ED_{i} \right| }{S_{i}} \end{aligned}$$

12 $$\begin{aligned} NMED= & \frac{1}{2^{2M}\left( 2^{M} - 1\right) ^{2}}\sum _{i=1}^{2^{2M}}\left| ED_{i} \right| \end{aligned}$$

MRED is defined by dividing the precise output of the ED between the approximation and exact output for each combination of input operands like in Eq. (11).Where M is the number of bits of a multiplier, $S_{i}$ is a precise outcome for every input-operand combination. The NMED metric, which represents the mean error distance normalized by the maximum error value that an inaccurate multiplier can have, is calculated as follows as in Eq. (12):

Table 9 details the precision metrics for the multipliers. The data was derived by evaluating all 65,536 inputs for the 8-bit multipliers. However, in applications where precision is less critical, parameters related to error distance become more important. The newly proposed multiplier shows a lower Mean Relative Error Distance (MRED) than the multiplier created with the compressor mentioned in Table 9. Figure 22 shows the MRED of the proposed multiplier with the existing compressors. displays impressive accuracy with MRED (Mean Relative Error Distance) values of 0.0304 at 0.5V and 1V.

The proposed design provides a strong trade-off between energy efficiency and computational quality. With a MRED of $3.04 \times 10^{-2}$ and NMED of 0.0409, the design performs on par with or better than recent CMOS-based architectures. It also records a 1-NMED of 0.9591, confirming its suitability for quality-constrained applications. Comparative figures such as DQ4:$2C_{2}$^12^ show MRED of 0.2932 and NMED of 0.037, which result in higher error spread and less stable output.

When compared to other multipliers, it shows considerable improvement. For instance, it demonstrates 41.6% and 56.5% higher accuracy than^26^, with MRED values of 0.0521 and 0.0699, respectively. It also surpasses^13^ by over 99%, with MRED values of 4.5483 and 4.2843. Furthermore, it offers 91.8% better accuracy than^12^, with an MRED of 0.3692. Even in cases where the differences are more modest, such as with^16^, which has an MRED of 0.0326, the proposed multiplier shows a 6.7% improvement. Compared to^25^, with an MRED of 0.0292, the proposed multiplier exhibits a slight increase in accuracy by about 4.1%. Additionally, compared to^14^, which has an MRED of 1.2, it shows a significant improvement of 97.5%. Finally, compared to^15^, with an MRED of 0.3002, the proposed multiplier achieves an 89.9% higher accuracy. MRED and PDP are considered to evaluate the accuracy and energy efficiency of the designed multiplier. Fig.23 presents the PDP $\times$ MRED for all the 8*8 multipliers. At 0.5V, the proposed 8*8 multiplier achieves a PDP $\times$ MRED value of 0.217, significantly lower than most other references. For example, it is about 75.8% and 78.3% lower than^26^, over 99% lower than^13^ and between 97.4% and 99.3% lower compared to the various values of^12^. At 1V, the proposed multiplier achieves a value of 0.949, which remains better than most references, including a reduction of 99% compared to^13^ and 91.8% compared to^12^. However, it is slightly higher than^24^ by about 15.8%. This analysis underscores the proposed multiplier’s exceptional energy efficiency and accuracy performance across various conditions.

### Application: image multiplication

In this section, we assess the practical utility of imprecise multipliers by applying them to image multiplication–an essential operation in image processing. A MATLAB program has been deployed to perform pixelwise multiplication using the described approximate multipliers. Several factors, including PSNR and MSSIM^31^, must be considered when assessing image quality. The PSNR, a widely recognized metric for assessing image quality , is computed to evaluate the precision of the output images. While SSIM measures the structural similarity of two images concerning the human visual system.

Eight pairs of standard images were chosen for image multiplication^30^: (Cameraman, moon surface), (clock, airplane), (house, Rice), (Mandrill, Peppers), (football, Cameraman), (cameraman,sky),(sky,moon) and (Jet, house). Each pair was multiplied, and the resulting images were analyzed to assess efficiency. Figures 24 and 26 shows all eight pairs of images with their exact and approximate output images. Table 10 shows the PSNR and MSSIM values for all eight pairs.

The provided MSSIM values are illustrated in Table 11 how different multiplier architectures maintain structural integrity during image multiplication. The proposed approach achieves outstanding MSSIM scores of 0.9469 for the Cameraman * Moon Surface pair and 0.9767 for the Clock * Airplane pair. It consistently outperforms other benchmarks, indicating enhanced image quality. For instance, MSSIM values from^13^ are 0.65 and 0.68, along with 0.6642 and 0.6971 for the respective image pairs, showcasing significantly lower preservation of structural similarity. Even the higher scores from^16^, at 0.93 and 0.952, do not reach the proposed work’s results. Additionally, references^7,14,15,24^ reveal varying image quality levels, yet none achieve the high MSSIM scores of the proposed multipliers. This comparison highlights the proposed multipliers’ effectiveness in achieving superior structural fidelity, rendering them exceptionally efficient for image processing tasks where preserving image quality is crucial. Table 12 provides a comprehensive evaluation of PSNR and MSSIM scores for several 8*8 approximate multipliers tested on three standard image pairs. The proposed multiplier exhibits clearly superior performance across all metrics. Specifically, it achieves a PSNR of 45.35dB and MSSIM of 0.98 for the Sky * Moon pair, indicating strong visual fidelity. In all tested scenarios, the proposed design maintains MSSIM values exceeding 0.98, demonstrating high structural similarity with ground-truth results. In contrast, previously reported designs such as^13^ and^12^ show significantly lower PSNR and MSSIM values, pointing to greater visual distortion. These results emphasize the effectiveness of our design in preserving image quality, making it well-suited for low-power, error-tolerant multimedia processing tasks.

To further validate the visual fidelity of the proposed approximate multiplier, error maps and heat maps were generated for eight standard image pairs is shown in Figs. 25 and 27. The error maps, created by pixel-wise difference between exact and approximate outputs, remain largely dark across all cases, indicating minimal deviations. Similarly, the corresponding heat maps show mostly low-intensity regions, reinforcing that the approximation introduces only localized, low-magnitude changes.These visual comparisons provide qualitative evidence aligned with the PSNR and MSSIM values presented in Tables 10 and 12, demonstrating that the proposed multiplier achieves strong image quality retention across various test scenarios. Especially in images with complex textures (such as Mandrill and Jet), the approximate outputs remain visually close to the exact results, with only slight variations visible in the error and heat map representations. Thus, the error and heat map visualizations confirm that the proposed design achieves high accuracy while preserving critical visual quality in approximate image processing tasks.

## Conclusion

The proposed work highlights significant improvements in image multiplication and overall performance metrics compared to current available architectures. The suggested compressor, operating at 1V and 0.5V, showcased remarkable delay and energy efficiency with delays of 10ps and 4ps, and power consumptions of 0.269$\mu$W and 0.03$\mu$W, simulated using NCFET 45nm technology in Cadence Virtuoso. The 8*8 multipliers at both voltage levels illustrated the design’s efficiency, displaying low PDP of 58.3% and EDP of 4.104(fJ$\cdot$ns)values. Accuracy metrics such as MED, MRED, and NMED indicated high precision. Meanwhile, the PSNR and MSSIM values for image pairs (Cameraman * Moon Surface and Clock * Airplane) reached outstanding levels at 55.8 and 0.9469 and 41.5 and 0.9767, respectively. The proposed approach consistently yielded superior MSSIM values compared to other reference points, underscoring its high image quality and structural integrity. These results confirm the superior effectiveness of the proposed multipliers in maintaining image quality, making them particularly efficient for image processing applications.

## Data Availability

The dataset was gathered from the publicly available, which is available online. It is publicly accessible and unrestricted “SIPI.” USC. 2022. [Online]. Available: https://sipi.usc.edu/database/.
